# Kinetics of the Reaction Between the Criegee Intermediate CH_2_OO and NO_2_: Experimental Measurements and Comparison with Theory

**DOI:** 10.1021/acs.jpca.4c08203

**Published:** 2025-02-12

**Authors:** Rachel E. Lade, Kate A. Livesey, Luc Vereecken, Robin J. Shannon, Mark A. Blitz, Paul W. Seakins, Daniel Stone

**Affiliations:** 1 School of Chemistry, University of Leeds, Leeds LS2 9JT, U.K.; 2 Institute for Energy and Climate Research, ICE-3: Troposphere, Forschungszentrum Jülich GmbH, Jülich 52425, Germany; 3 National Centre for Atmospheric Science, University of Leeds, Leeds LS2 9JT, U.K.

## Abstract

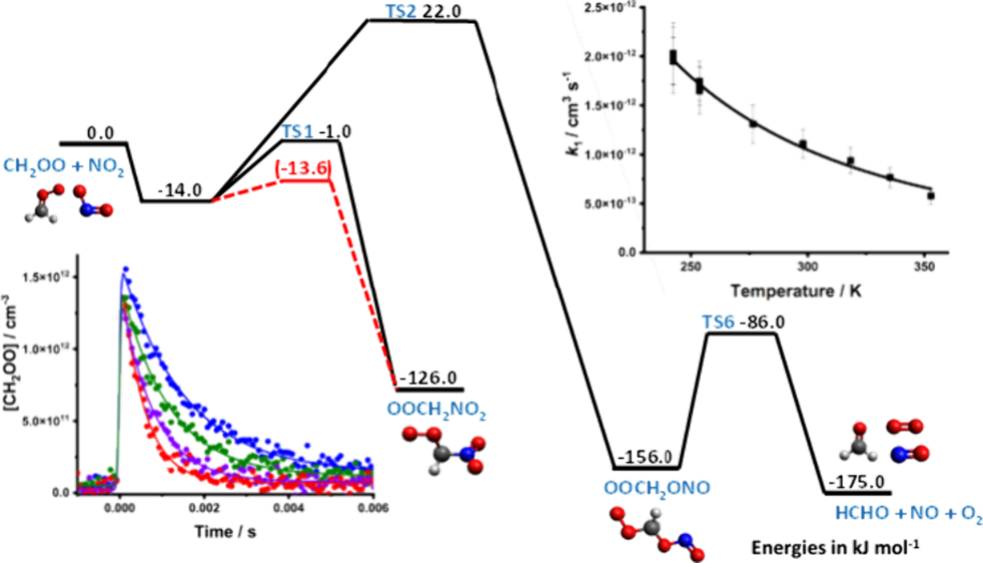

Kinetics of the gas phase reaction between the stabilized Criegee intermediate formaldehyde oxide (CH_2_OO) and nitrogen dioxide (NO_2_) have been measured using laser flash photolysis of CH_2_I_2_/O_2_/N_2_/NO_2_ mixtures coupled with time-resolved broadband ultraviolet absorption spectroscopy. Experiments were performed in N_2_ under pseudo-first-order conditions at temperatures between 242 and 353 K and pressures in the range 25 to 300 Torr. The kinetics of CH_2_OO + NO_2_ are independent of pressure, with a mean rate coefficient of *k*_1_ = (1.24 ± 0.22) × 10^–12^ cm^3^ s^–1^ at 298 K, where the uncertainty represents a combination of the 1σ statistical error and the systematic errors resulting from uncertainties in gas flow rates and in the concentration of NO_2_. Measurements indicate upper limits of <5% for production of NO_3_ and <5% for production of NO, and further studies of product yields are warranted. In contrast to expectations from theory, the kinetics of CH_2_OO + NO_2_ display a negative temperature dependence that can be described by *k*_1_ = (1.07 ± 0.02) × 10^–12^ × (*T*/298)^−(2.9±0.2)^ cm^3^ s^–1^. Analysis using the Master Equation Solver for Multi-Energy well Reactions is able to reproduce a negative temperature dependence for the reaction if significant changes to barrier heights are made, but the overall agreement between the experiment and theory remains poor. This work highlights the challenges associated with calculations for systems with significant multi-reference character.

## Introduction

Gas phase oxidation processes control the lifetimes of many trace species emitted into the atmosphere, and thus their impacts on climate, air quality, and human health. Oxidation processes initiated by ozone (O_3_) lead to the production of zwitterionic Criegee intermediates (R_2_COO), which have high internal energy on initial production and may undergo rapid decomposition or stabilization through collisions with surrounding bath gases.^1^ The chemistry of stabilized Criegee intermediates has the potential to impact atmospheric oxidation capacity through unimolecular decomposition processes, which can result in production of key species such as hydroxyl radicals (OH), or bimolecular reactions with species such as water, water dimers, organic acids, SO_2_, and NO_2_.^2^

The reaction of formaldehyde oxide (CH_2_OO), the simplest Criegee intermediate, with NO_2_ (reaction R1) has been the subject of a number of studies, but there are significant discrepancies in the kinetics at room temperature, uncertainties relating to the nature of products, and thus far, there have been no experimental measurements of the temperature dependence of the kinetics.
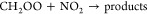
R1

The first direct measurements of the kinetics of reaction R1 were made by Welz et al.^3^ at 4 Torr and 298 K using laser flash photolysis of CH_2_I_2_ at λ = 248 nm in the presence of O_2_ to generate CH_2_OO, with detection of the Criegee intermediate using photoionization mass spectrometry (PIMS). Experiments were performed using ^13^CH_2_OO to enable separation of the CH_2_OO signal from that of ^14^NO_2_, with results indicating *k*_1_ = (7_–2_^+3^) × 10^–12^ cm^3^ s^–1^.

The potential for pressure dependence of *k*_1_ was subsequently investigated by Stone et al.^4^ at 295 K and pressures between 25 and 300 Torr. Laser flash photolysis of CH_2_I_2_/O_2_ at λ = 248 nm was again used to generate CH_2_OO, which produces CH_2_OO in high yield at low pressures but leads to significant production of the peroxy radical CH_2_IO_2_ instead of CH_2_OO at higher pressures (reactions R2–R3):
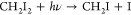
R2
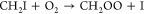
R3a
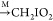
R3b

The kinetics of reaction R1 were determined using laser-induced fluorescence (LIF) spectroscopy to monitor formaldehyde (HCHO) production in the system, which was produced from both CH_2_OO and CH_2_IO_2_ in the absence of NO_2_. In the presence of NO_2_, HCHO was produced from CH_2_OO only, with inhibition of HCHO production from CH_2_IO_2_ owing to production of the peroxy nitrate CH_2_IO_2_NO_2_. Experiments gave a mean value of *k*_1_ = (1.5 ± 0.5) × 10^–12^ cm^3^ s^–1^ which displayed no significant dependence on pressure over the range investigated, and yields of CH_2_OO and CH_2_IO_2_ from reaction R3 determined from measurements of HCHO in experiments using CH_2_I_2_/O_2_/N_2_/NO_2_ were in agreement with those determined via other methods.^5,6^

Measurements of *k*_1_ at room temperature have also been made at low pressure using laser flash photolysis of CH_2_I_2_/O_2_ and direct detection of CH_2_OO by mid-infrared absorption spectroscopy with QCL lasers. Qiu and Tonokura^7^ monitored CH_2_OO via its absorption in the region 1273.0–1277.5 cm^–1^ following production using λ = 266 nm and reported a value for *k*_1_ of (4.4 ± 0.2) × 10^–12^ cm^3^ s^–1^ at 10 Torr and 295 K, while Luo et al.^8^ monitored CH_2_OO via absorption in the region 880–932 cm^–1^, with production using λ = 248 nm, and reported *k*_1_ = (1.0 ± 0.2) × 10^–12^ cm^3^ s^–1^ at pressures in the range 6–10 Torr at 298 K.

Previous measurements of *k*_1_ are summarized in Table 1. Kinetics reported by Stone et al.^4^ and Luo et al.^8^ are in agreement, whereas Qiu and Tonokura^7^ and Welz et al.^3^ report higher values. The study by Qiu and Tonokura employed relatively high initial concentrations of CH_2_OO (∼10^14^ cm^–3^) and although the analysis considered self-reaction of the Criegee intermediate the results may have been impacted by other secondary chemistry, including potential reaction with products resulting from the reaction of CH_2_OO with NO_2_. The current 298 K IUPAC recommended value for *k*_1_ is (3_–2_^+6^) × 10^–12^ cm^3^ s^–1^.^9^

**Table 1:** Summary of Results for *k*_1_ Obtained in This and Previous Work^a^

temperature (K)	pressure (Torr)	*k*_1_ (10^–12^ cm^3^ s^–1^)	reference
298	4	7. 0_–2_^+3^	Welz et al.^3^
295	25–300	1.5 ± 0.5	Stone et al.^4^
295	10.4	4.4 ± 0.2	Qiu and Tonokura^7^
298	5.9–9.7	1.0 ± 0.2	Luo et al.^8^
242	25	1.96 ± 0.34	this work
	50	1.95 ± 0.24	
	200	2.03 ± 0.31	
254	25	1.65 ± 0.24	
	50	1.70 ± 0.20	
	200	1.75 ± 0.20	
277	50	1.31 ± 0.19	
298	25	1.19 ± 0.14	
	50	1.11 ± 0.14	
	100	1.24 ± 0.15	
	200	1.39 ± 0.15	
	300	1.28 ± 0.15	
318	50	0.94 ± 0.13	
335	50	0.77 ± 0.10	
353	50	0.58 ± 0.08	

a The uncertainties in this work represent a combination of the 1σ statistical error and the systematic errors resulting from uncertainties in gas flow rates and in the concentration of NO_2_.

The experiments performed by Stone et al.^4^ indicated that HCHO is produced in the system, and although NO_3_ has been reported as a product by Ouyang et al.,^10^ the measurements of NO_3_ were not direct or on the time scale of the reaction. Subsequent experiments have indicated that any NO_3_ observed was a result of secondary chemistry involving iodine species (e.g., INO_2_ + IONO_2_ → NO_3_ + NO_2_ + I_2_) in the system.^11^ Products of reaction R1 have also been investigated by Caravan et al.^12^ using the PIMS technique following photolysis of CH_2_I_2_/O_2_ at λ = 248 nm. A mass signal consistent with the formation of an adduct between CH_2_OO and NO_2_ was identified, but production of NO_3_ was not observed.

**Figure 1:**
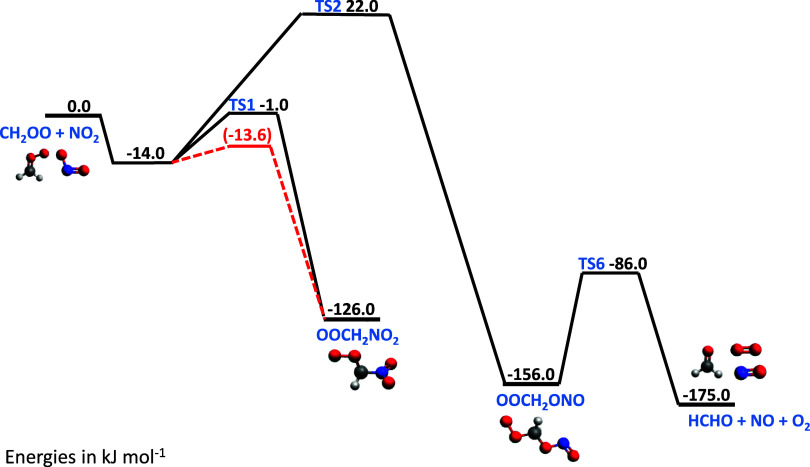
Simplified potential energy surface for the reaction between CH_2_OO + NO_2_ based on the results reported by Vereecken and Nguyen. Solid lines and values in black show the surface reported by Vereecken and Nguyen.^14^ Dashed lines and values in red show the result obtained by fitting the barrier height for TS1 to the experimental observations made in this work using MESMER.

The potential energy surface for reaction R1 has been calculated by Presto and Donahue^13^ at the B3LYP/6-31G(d,p) level of theory, and by Vereecken and Nguyen^14^ at the CCSD(T)/aug-cc-pVTZ//M06-2X level of theory, with certain key transition states also investigated at higher levels of theory, including multi-reference NEVPT2/aug-cc-pVTZ. The calculations reported by Vereecken and Nguyen indicate two reaction channels relevant to atmospheric conditions shown in Figure 1, both of which involve the initial barrierless production of a pre-reaction complex. The reaction channel with the lowest energy transition state leads to the production of an adduct between CH_2_OO and NO_2_ which is bound via a C–N bond and is stable with respect to unimolecular isomerization or decomposition under atmospheric conditions. However, the adduct might react in a similar manner to peroxy radicals with species such as NO or other peroxy radicals to produce OCH_2_NO_2_, which is expected to decompose rapidly to produce HCHO and NO_2_. The other reaction channel operating under atmospheric conditions leads to the production of an adduct between CH_2_OO and NO_2_ which is bound via a C–O bond and is expected to dissociate rapidly to produce HCHO, O_2_, and NO. The relative importance of the two channels is subject to significant uncertainty owing to challenges associated with multi-reference effects, leading to estimated uncertainties in the transition state energies of ∼20 kJ mol^–1^.^14^ Kinetics of reaction R1 predicted from the calculated potential energy surface indicated *k*_1_ = 4.4 × 10^–12^ cm^3^ s^–1^ at 298 K and an overall weak positive temperature dependence between 200 and 400 K described by *k*_1_ = 1.15 × 10^–11^ exp(−278/*T*) cm^3^ s^–1^.

Experimental measurements of the temperature dependence of *k*_1_ have yet to be reported in the literature, and there are significant discrepancies between measurements made at room temperature and uncertainties in the nature of the reaction products of reaction R1. Laser flash photolysis of CH_2_I_2_/O_2_/N_2_/NO_2_ mixtures coupled with time-resolved broadband UV absorption spectroscopy has been used in this work to measure the kinetics of reaction R1 at temperatures between 242 and 353 K and pressures in the range 25 to 300 Torr.

## Experimental Section

Kinetics of the reaction between CH_2_OO and NO_2_ were investigated under pseudo-first-order conditions, with NO_2_ present in excess over CH_2_OO, using laser flash photolysis of CH_2_I_2_/O_2_/N_2_/NO_2_ mixtures coupled with time-resolved broadband UV absorption spectroscopy. The experimental apparatus has been described in detail in previous work^15,16^ and only a brief description is given here.

Gases N_2_ (BOC, 99.998%) and O_2_ (BOC, 99.5%) were mixed in a gas manifold with dilute mixtures of NO_2_ (Sigma-Aldrich, 99.9%), prepared manometrically in N_2_ (BOC, 99.998%), at known flows controlled by calibrated mass flow controllers. The precursor CH_2_I_2_ (Alfa Aesar, 99%) was introduced into the flow by passing a small fraction of the flow, controlled by a needle valve, through a bubbler containing liquid CH_2_I_2_ at a fixed temperature in a water-ice bath which was recombined with the main flow prior to entry into the reaction cell. The concentrations of CH_2_I_2_ and NO_2_ were measured periodically by UV absorption spectroscopy. Typical precursor concentrations were (4.0–7.5) × 10^13^ cm^–3^ CH_2_I_2_, (0.4–1.5) × 10^18^ cm^–3^ O_2_, and (0.09–1.43) × 10^15^ cm^–3^ NO_2_. Relative concentrations of O_2_ and NO_2_ were selected to minimize impacts of the reaction of CH_2_I with NO_2_.

The reaction cell, which was sealed with fused silica windows at each end and had a length of 100 cm and diameter of 3 cm, was jacketed to allow temperature control of the gas within the cell via the flow of liquid from a recirculating thermostatting unit (Huber Unistat 360) through the outer jacket. The temperature was calibrated in separate experiments under identical flow conditions in the cell which have been described previously.^15,17^ Pressure within the cell was measured by a capacitance manometer (MKS Instruments) and controlled by throttling the exit to the cell to a rotary pump (EM2, Edwards). The total flow rate through the cell was set at 1200 standard cm^3^ per minute (sccm) at 298 K and 50 Torr and adjusted with temperature and pressure to maintain a constant residence time in the cell of ∼3 s.

The Criegee intermediate CH_2_OO was generated within the cell by reactions R2–R3. Photolysis of CH_2_I_2_ (reaction R2) was initiated by an excimer laser operating at λ = 248 nm with typical fluence of 20–30 mJ cm^–2^ (KrF, Lambda-Physik CompEx 210) which was aligned along the length of the reaction cell using a dichroic turning mirror (Edmund Optics), giving typical initial CH_2_OO concentrations of (1.1–2.5) × 10^12^ cm^–3^.

The probe light was provided by a laser-driven light source (LDLS, Energetiq EQ-99X), which provided ∼10 mW cm^–2^ of light with near constant radiance across the spectral range 200–800 nm. The probe beam was collimated by an off-axis parabolic mirror (ThorLabs) and passed through the reaction cell seven times by a series of Al mirrors (12 mm diameter, Knight Optical), leading to an effective path length of (471 ± 50) cm which was measured using the method described in our previous work.^15^

The probe beam was passed through a sharp cut-on filter (248 nm RazorEdge ultrasteep long-pass edge filter) to reduce the impact of scattered light from the photolysis laser and focused into a fiber optic via a fiber launcher (Elliot Scientific). Output from the fiber optic was imaged through a 25 μm slit onto an integrated spectrograph (600 grooves/mm) and charge-coupled device (CCD) detector (FER-SCI-1024BRX, Princeton Instruments). Light intensities were measured on an illuminated region of the CCD detector, with spectral resolution of 1.1 nm. Temporal resolution between 90 and 400 μs was achieved by the periodic transfer of photocharge from the illuminated region of the CCD to a storage region shielded from incoming radiation at set time intervals throughout the reaction. Synchronisation of the CCD detector and photolysis laser was controlled by a delay generator (SRS DG535), which was operated with a pulse repetition frequency of 0.25 Hz to ensure that the gas mixture in the cell was replaced between each photolysis shot. Intensity data were typically recorded for 300 photolysis shots and transferred to a PC for analysis.

Experiments using laser-induced fluorescence (LIF) spectroscopy to investigate potential production of NO are described in the Supporting Information (Section S7).

## Theoretical Calculations

The Master Equation Solver for Multi-Energy well Reactions (MESMER) was used to explore the sensitivity of calculated rate coefficients to the potential energy surface for reaction R1. MESMER uses an energy-grained master equation approach which has been described in detail in previous work,^18^ and can be used to optimize potential energy surfaces, including transition state energies, to fit to experimental results.

Geometries, vibrational frequencies, and rotational constants required as inputs for each species considered were obtained from calculations performed at the M06-2X/cc-pVTZ level of theory in Gaussian09 using the optimized geometries reported by Vereecken and Nguyen as the initial structure for each species. Hindered rotation potentials for the transition states were obtained from M06-2*X*/6-31+G** relaxed scans along the relevant dihedral coordinates. Hindered rotor state densities were calculated in MESMER using the methodology described in previous work.^19^ It should be noted that the intention of the electronic structure and hindered rotor calculations was not to attempt improvements to the work of Vereecken and Nguyen, as the calculations in this work were performed at a lower level of theory, but to provide appropriate inputs with a physical basis for the MESMER calculations.

Rate coefficients were calculated in MESMER using an Inverse Laplace Transform (ILT) for the initial barrierless formation of the pre-reaction complex, with the rate coefficient described by *k* = *AT*^*n*^, and RRKM theory for other reactions. A rigid rotor-harmonic oscillator approximation was made for all but the hindered modes, which were assumed to be separable. Lennard-Jones parameters used to describe collisions with the bath gas were estimated from work by Vereecken et al.,^20^ with collisional energy transfer described by an exponential down model in which the average energy transferred in a downward direction on collision was represented by the parameter ⟨Δ*E*⟩_down_. For calculations reported in this work, a value of 250 cm^–1^ was used for ⟨Δ*E*⟩_down_ which was approximated as being independent of temperature owing to the relatively narrow range of temperatures considered. It is noted that neither the experiments, nor the preliminary MESMER calculations, suggest any pressure dependence in this system and the calculated rates are therefore insensitive to the collisional energy transfer parameters. The input file for MESMER is provided in the Supporting Information (Section S12).

## Results and Discussion

Measured intensities were converted to absorbance spectra, which were then used to determine concentrations of each absorbing species by fitting reference absorption cross-sections using the Beer–Lambert law (eq 1):
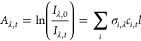
1where *A*_λ, *t*_ is the total absorbance at wavelength λ and time *t*, *I*_λ,0_ is the average pre-photolysis light intensity at wavelength λ, *I*_λ, *t*_ is the post-photolysis light intensity at wavelength λ and time *t*, σ_*i*, λ_ is absorption cross-section of species *i* at wavelength λ, *c*_*i*, *t*_ is the concentration of species *i* at time *t*, and *l* is the effective path length, which has a value of (471 ± 50) cm for experiments reported in this work.

**Figure 2:**
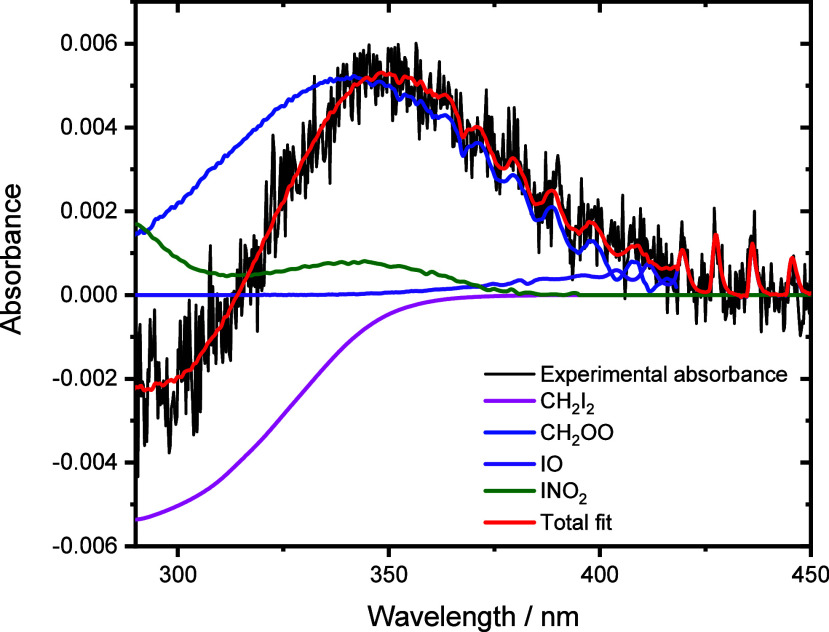
Typical observed absorbance (black) and total fit (red) obtained by fitting reference spectra for CH_2_I_2_ (pink),^30^ CH_2_OO (blue),^22^ INO_2_ (green),^31^ and IO (purple)^32^ using eq 1. Data shown were obtained at 1 ms after photolysis at *p* = 100 Torr and *T* = 298 K, with [CH_2_I_2_] = 5.9 × 10^13^ cm^–3^, [O_2_] = 4.0 × 10^17^ cm^–3^, and [NO_2_] = 8.1 × 10^14^ cm^–3^. The fit gave Δ[CH_2_I_2_] = 3.0 × 10^12^ cm^–3^, [CH_2_OO] = 8.0 × 10^11^ cm^–3^, [IO] = 5.8 × 10^10^ cm^–3^, and [INO_2_] = 2.0 × 10^12^ cm^–3^.

Figure 2 shows a typical absorbance spectrum obtained following photolysis and the fit to the total absorbance obtained by fitting reference cross sections for absorbing species present. In the absence of NO_2_, the main absorbing species were the precursor CH_2_I_2_, the Criegee intermediate CH_2_OO, and IO radicals which are generated via secondary chemistry within the system^21,22^ (see the Supporting Information, Sections S1 and S2). In the presence of NO_2_, IO radicals were observed in lower concentrations, but there was production of INO_2_, which absorbs in the wavelength region of interest for CH_2_OO, and I_2_ was also observed. There was no evidence for production of NO_3_, and an upper limit of 5% can be placed on the yield of NO_3_. Experiments using LIF spectroscopy to investigate potential production of NO indicate an upper limit of 5% on the yield of NO. Further details are given in the Supporting Information (Sections S3 and S7).

**Figure 3:**
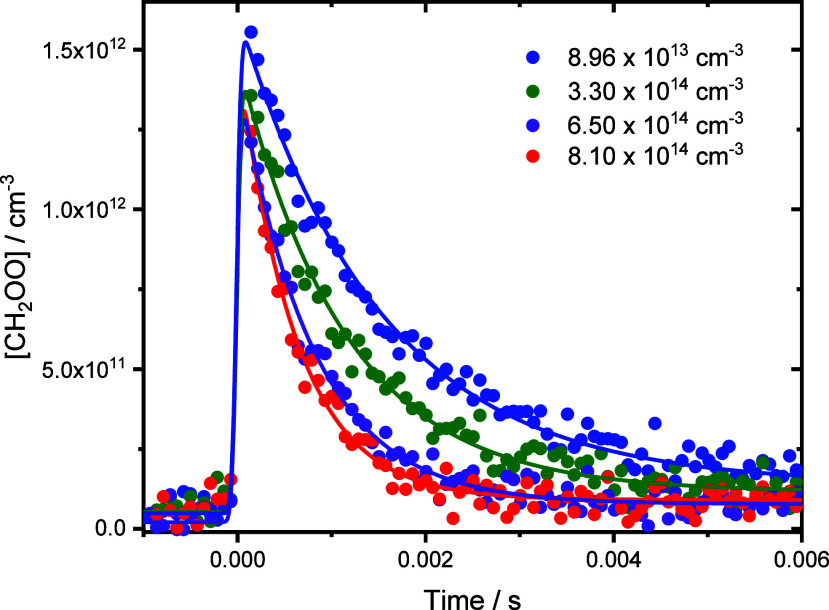
Concentration–time profiles for CH_2_OO in the presence of NO_2_. For these experiments, *p* = 100 Torr and *T* = 298 K. Solid lines represent unweighted fits to eq 2 convoluted with the instrument response function (see the Supporting Information for further details). For [NO_2_] = 8.96 × 10^13^ cm^–3^ (blue data points), the fit gave *k′* = (666 ± 18) s^–1^; for [NO_2_] = 3.30 × 10^14^ cm^–3^ (green data points), the fit gave *k′* = (885 ± 27) s^–1^; for [NO_2_] = 6.50 × 10^14^ cm^–3^ (purple data points), the fit gave *k′* = (1271 ± 44) s^–1^; and for [NO_2_] = 8.10 × 10^14^ cm^–3^ (red data points), the fit gave *k′* = (1642 ± 62) s^–1^. The instrument response parameters were *t*_c_ = −(1.05 ± 0.13) × 10^–5^ s and *w* = (4.05 ± 0.90) × 10^–5^ s (see the Supporting Information for further details). Uncertainties are statistical at the 1σ level. Fits to the data using a mixed first- and second-order model are shown in the Supporting Information, Section S5.

**Figure 4:**
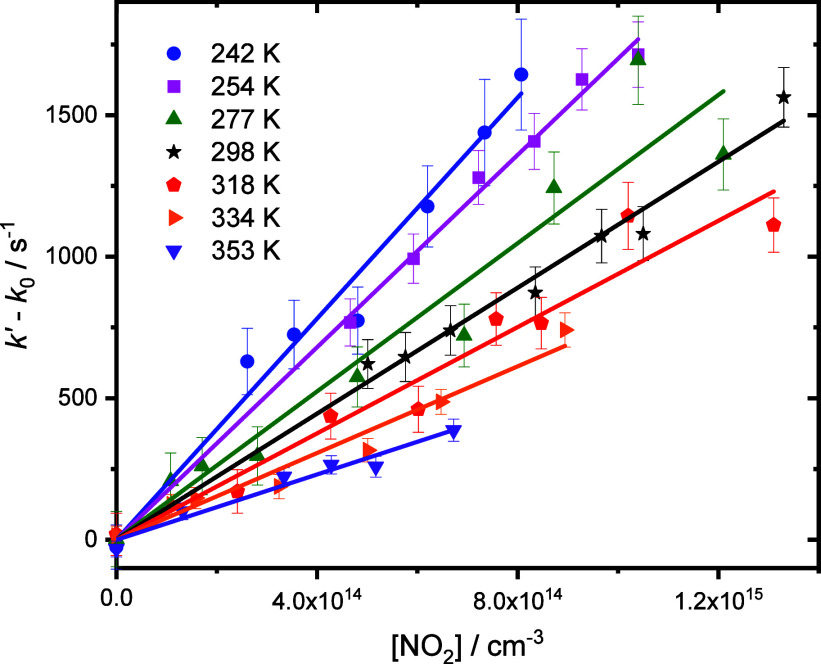
Dependence of pseudo-first-order rate coefficients on the concentration of NO_2_ at each temperature investigated in this work at *p* = 50 Torr. For clarity, rate coefficients obtained in the absence of NO_2_ (*k*_0_, with typical values of ∼400 s^–1^) have been subtracted from the pseudo-first-order rate coefficients (*k*′) obtained from fits of concentration–time profiles (Figure 3) to eq 2. Slopes of the plots at each temperature are used to determine *k*_1_. Uncertainties shown are statistical at the 1σ level from the fits to decay traces.

Figure 3 shows a typical concentration–time profile for CH_2_OO. Kinetics describing the loss of CH_2_OO were determined by fitting eq 2, convoluted with an instrument response function (IRF) (see the Supporting Information, Section S4 for further details), to each concentration–time profile.
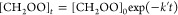
2where [CH_2_OO]*_t_* is the concentration of CH_2_OO at time *t*, [CH_2_OO]_0_ is the initial concentration of CH_2_OO, and *k*′ is the rate coefficient describing the sum of first-order losses of the CH_2_OO conformer and is given by *k*′ = *k*_0_ + *k*_1_[NO_2_], where *k*_0_ represents losses of CH_2_OO by any reaction or process other than reaction with NO_2_. The rate coefficients *k*_1_ were determined from the slopes of plots of *k*′ against the NO_2_ concentration, as shown in Figure 4.

**Figure 5:**
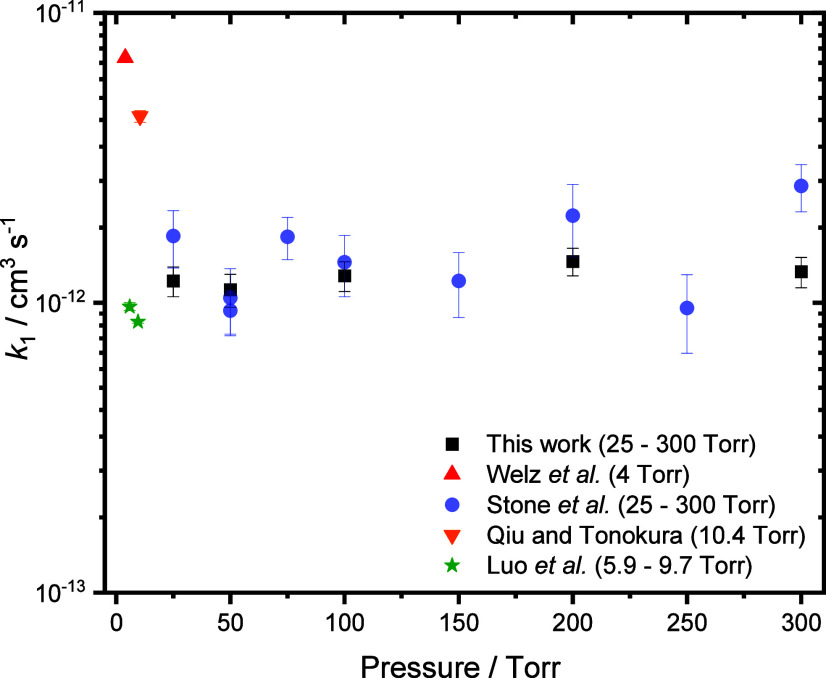
Summary of results for *k*_1_ obtained at ∼298 K in this work (black squares) and in previous work by Welz et al. (red triangle),^3^ Stone et al. (purple circles),^4^ Qiu and Tonokura (orange inverted triangle),^7^ and Luo et al. (green stars).^8^ The uncertainties represent a combination of the 1σ statistical error and the systematic errors resulting from uncertainties in gas flow rates and in the concentration of NO_2_.

Results at 298 K are shown in Figure 5 and summarized in Table 1, and indicate that there is no significant dependence of *k*_1_ on pressure over the range investigated, in agreement with previous work^4^ but with a significant improvement in precision. Potential effects of pressure were also investigated at 242 and 254 K, with no significant dependence on pressure observed at either temperature (see the Supporting Information, Section S6). At 298 K, a mean value for *k*_1_ of (1.24 ± 0.22) × 10^–12^ cm^3^ s^–1^ was obtained, where the uncertainty represents a combination of the 1σ statistical error and the systematic errors resulting from uncertainties in gas flow rates and in the concentration of NO_2_, in agreement with previous measurements by Stone et al.^4^ and Luo et al.^8^ Measurements of *k*_1_ reported by Welz et al.^3^ and Qiu and Tonokura^7^ are higher than those determined in this work by as much as a factor of 6, although both Welz et al. and Qiu and Tonokura used higher initial concentrations of CH_2_OO which may have promoted secondary chemistry and impacted results.

**Figure 6:**
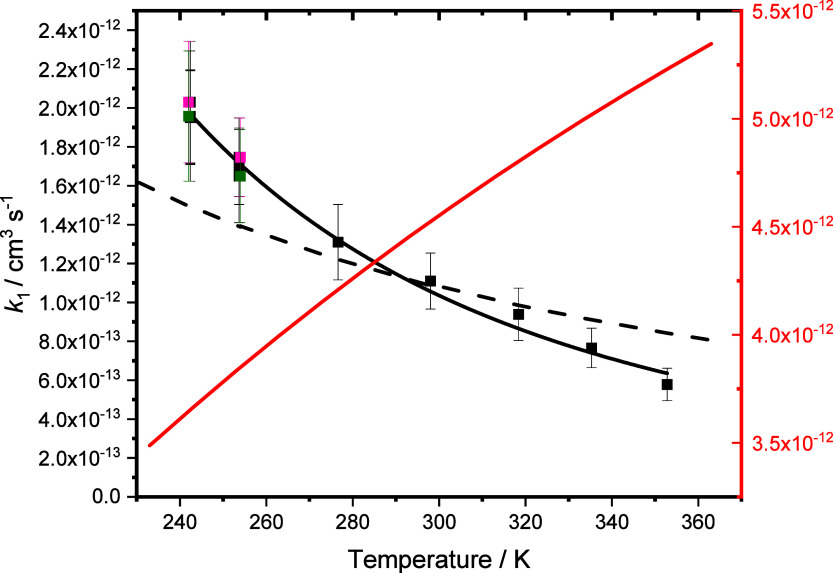
Effects of temperature on *k*_1_ observed in this work at 25 Torr (green), 50 Torr (black), and 200 Torr (pink), predicted by Vereecken and Nguyen (red),^14^ and fit using MESMER by varying energy of TS1 (black dashed line). Experimental results obtained in this work are parametrized by *k*_1_ = ((1.07 ± 0.02) × 10^–12^) × (*T*/298)^−2.9±0.1^ cm^3^ s^–1^ (black solid line). Uncertainties are 1σ.

Figure 6 shows the effects of temperature on *k*_1_, with results summarized in Table 1. In contrast to expectations from theory, which predicted a positive temperature dependence, the observations reveal a negative temperature dependence that is well described by the expression *k*_1_ = (1.06 ± 0.02) × 10^–12^ × (*T*/298)^−(2.9±0.2)^ cm^3^ s^–1^. The observed temperature dependence suggests a barrierless reaction, with the relatively slow reaction indicating a submerged barrier leading from initial barrierless formation of a pre-reaction complex that has a significant entropic barrier to product formation. Thus, while the barrierless formation of the pre-reaction complex may be rapid and give rise to a negative temperature dependence, the forward reaction from the pre-reaction complex to products occurs with a tight transition state that is significantly slower than the reverse reaction back to reagents via a loose transition state.

Barrierless reactions have been observed for other reactions of CH_2_OO, including those with water dimers,^23,24^ SO_2_,^15^ and formic acid.^25^ While there has been prediction of positive temperature dependence for reactions of the Criegee intermediates CH_2_OO, *syn*-CH_3_CHOO, and (CH_3_)_2_COO with SO_2_,^26^ the observed negative temperature dependence for other reactions, including CH_2_OO + SO_2_, have been rationalized by theory in other studies.^20,27^ The discrepancy in barrier heights between experiment and theory for CH_2_OO + NO_2_ reflects the challenges associated with calculations involving transition states with significant multi-reference character.

Previous calculations by Vereecken and Nguyen^14^ indicated substantial multi-reference character in the entrance channel transition states, with single-reference methods overestimating the barrier heights by up to ∼20 kJ mol^–1^, where the energies for the lowest energy channels were found to be unusually sensitive to the size of the active space used. IRCMax calculations at the NEVPT2(15,14) level of theory along the M06-2X intrinsic reaction coordinate (IRC) path showed a negligible shift for the position with the highest energy along that path, providing no evidence for a strong bias in transition state geometry. However, the improved wave function when using multi-reference methods may allow for a different, even more optimal geometric path with energies below those predicted using a single-reference method. This may also affect the various entrance channels differently, and even change their relative importance. As such, considerable uncertainty remains regarding this portion of the potential energy surface. Unfortunately, Vereecken and Nguyen showed that a very large active space is necessary, requiring at least 11 orbitals with 11 electrons, and preferably as large as 15 orbitals with 15 electrons. As a consequence, transition state optimization, IRC calculations, and frequency calculations at a sufficiently high level of multi-reference theory are very expensive, and even then possibly still prone to errors due to inconsistencies in the active space throughout the optimization. It appears that the differences between experiment and theory must result from use of an insufficiently advanced level of theory, or the presence of an additional reaction channel which has not been identified at the level of theory used.

In this work, we opt to use a potential energy surface based on the available theoretical work but consider the properties of the potential energy surface entrance channels as adjustable parameters. Figure 1 shows the potential energy surface employed in MESMER, which considers only those reaction channels expected to contribute to the overall reaction under atmospheric conditions. MESMER calculations were fit to the experimentally determined values for *k*_1_ by varying the *A*-factor in the ILT description of the initial barrierless formation of the pre-reaction complex and, since we do not expect any significant reaction to occur via TS2 owing to the upper limits placed on the production of NO (see the Supporting Information, Section S7), the barrier height TS1. In our calculations, the energy for TS1 needed to be lowered considerably in order to fit the experimental data, with the fit requiring a decrease from −1 kJ mol^–1^ to −14 kJ mol^–1^ and giving uncertainties in the fitted energy of TS1 on order of tens of kJ mol^–1^. The fitted value for the ILT parameter *A*, describing the formation of the pre-reaction complex, was (1.2 ± 1.6) × 10^–12^ cm^3^ s^–1^. In all fits, the ILT parameter *n* was fixed to its minimum value of −1.49 achievable in the software to maintain numerical stability, in order to best capture the observed temperature dependence, though a value of *n* ∼ −2.5 would reproduce the temperature dependence more closely. Fits in which both TS1 and TS2 were varied, and in which only TS2 was varied (shown in the Supporting Information, Section S8) did not display any significant differences in the fitted rate coefficients or fit quality.

At 298 K, the MESMER fits gave *k*_1_ = 1.1 × 10^–12^ cm^3^ s^–1^, and there was no significant pressure dependence in the MESMER result, in agreement with the experimental results. However, while the fit agrees well at 298 K and, in contrast to the result reported by Vereecken and Nguyen, does give a negative temperature dependence, in general the agreement between the experimental measurements and the fit is only qualitative. Clearly there is substantial uncertainty regarding the transition state energies owing to the aforementioned multi-reference effects, although this has been partially circumvented through fitting the barriers to the experimental data. Given the submerged nature of the transition states in our fitted model, the theoretical rate coefficients are particularly sensitive to inaccuracies in the geometries and thus the vibrational frequencies of the transition states. Specifically, the rovibrational characteristics used here could be affected by the multi-reference character of the transition state and it should be noted that the calculations for the potential energy surface for reaction R1 are not yet at an asymptote when it comes to basis set and active space size. Additionally, a submerged channel has its kinetic bottleneck at larger reactant separations and requires a variational approach where the rovibrational characteristics are actually energy-specific and affect the energy-specific rate coefficients of the loose transition state. In addition, the lack of a full consideration of the coupling between hindered rotational modes in the current model will have an impact on the predicted state density, especially at the larger separations of a variational transition state. The combined uncertainties on the state density and hence the energy-specific rates makes it hard to ascertain which of the two channels included in the optimization are optimal. Fits in which the energy of only one of TS1 or TS2 was varied did not improve the fit quality (see the Supporting Information for further details). Our use of rovibrational characteristics obtained for the higher energy, geometrically tight, and thus rigid transition state geometries is likely to lead to a significant underestimation of the state densities and hence exaggerates the required lowering of energies to provide the desired rates, and biases the predicted temperature dependence. However, higher-level calculations using multi-reference methodologies with a larger active space across a variational trajectory and with accurate characterization of the lowest-energy degrees of freedom are prohibitively costly, and are outside the scope of the current work.

Results obtained in our earlier work,^4^ in which the kinetics of reaction R1 were determined at room temperature via observations of HCHO production, are in good agreement with those determined in this work through direct measurements of CH_2_OO, indicating the reliability of the HCHO measurements. However, LIF experiments to investigate production of NO (described in the Supporting Information, Section S7), which theory suggests as the coproduct of HCHO, indicate an upper limit of 5% to the yield of NO, indicating that the reaction is not proceeding via TS2. Work by Caravan et al.^12^ observed a mass signal consistent with adduct formation, suggesting the reaction proceeds via TS1. There is thus an apparent inconsistency between the observed production of HCHO in our earlier work,^4^ the lack of production of NO, the observations of adduct formation by Caravan et al.,^12^ and the results of the MESMER calculations reported in this work. It is possible that the potential energy surface used in the MESMER calculations is incomplete, owing to the challenges associated with the calculations described above, and that there is a reaction channel that produces HCHO as the dominant product, but with a coproduct other than NO or NO_3_, and formation of the adduct observed by Caravan et al.^12^ as a minor product, but at observable concentrations using the PIMS technique. However, the apparent differences between experimental results could also be rationalized if the adduct O_2_CH_2_NO_2_ observed by Caravan et al. is the main product, produced via TS1, but undergoes subsequent chemistry on rapid time scales to produce HCHO. Vereecken and Nguyen suggested that the adduct formed via TS1 is stable with respect to unimolecular decomposition but might be expected to react in a similar manner to a peroxy radical, generating OCH_2_NO_2_ in reactions with species such as NO or peroxy radicals, which would rapidly decompose to produce HCHO and NO_2_. While NO is not present initially in the system, photolysis of NO_2_ by the photolysis laser or the probe laser in experiments to monitor HCHO by LIF could lead to production of NO. A sufficiently rapid reaction of the O_2_CH_2_NO_2_ adduct formed in reaction R1 with NO could lead to near-complete conversion of CH_2_OO to HCHO, consistent with the kinetics and yields measured in our earlier work. Modeling of the system and experiments to monitor HCHO by LIF spectroscopy following photolysis of CH_2_I_2_/O_2_/N_2_/NO_2_ mixtures at λ = 266 nm and λ = 355 nm (shown in the Supporting Information, Section S9) indicate that a reaction of the O_2_CH_2_NO_2_ adduct that leads to the rapid production of HCHO could explain the differences between the results of our earlier work and the observations made by Caravan et al.^12^ On balance, we tentatively assign the major direct product of reaction R1 as the O_2_CH_2_NO_2_ adduct, and note the potential for rapid chemistry that leads to the production of OCH_2_NO_2_ and subsequently HCHO + NO_2_ in our previous work.^4^ However, significant uncertainties remain in the nature and yields of the reaction products, and discrepancies remain between experiment and theory.

Capabilities for accurate prediction of reaction kinetics are critical for many areas, including atmospheric chemistry, combustion, and astrochemistry, particularly when reactions of interest or conditions required present significant experimental challenges. The application of theoretical approaches to understand the chemistry of Criegee intermediates has gained significant attention in recent years owing to increased awareness of the potential role of Criegee intermediates in the atmosphere, and the use of theory has provided a basis for understanding the behavior and reactivity of Criegee intermediates. Notably, theory has also been used to help develop structure activity relationships (SARs) for Criegee intermediate reactions^28,29^ and reaction conditions that have yet to be studied experimentally. If the results of SARs and predictions based on theoretical approaches are to be used in numerical models to evaluate atmospheric composition for applications relating to air quality and climate, it is essential that such approaches are reliable. This work highlights a significant discrepancy between experimental measurements and theory, indicating a continued need for experimental measurements, both for direct application and for providing a means to test the validity of theoretical approaches, as well as care when applying theory, particularly when reaction systems have significant multi-reference character.

## Conclusions

This work has measured the kinetics of the reaction between the simplest Criegee intermediate CH_2_OO and NO_2_ at temperatures between 242 and 353 K and pressures in the range 25 to 300 Torr. Experimental measurements show that the kinetics are independent of pressure, over the range investigated, with a mean value of (1.24 ± 0.22) × 10^–12^ cm^3^ s^–1^ at 298 K and a negative temperature dependence described by (1.06 ± 0.02) × 10^–12^ × (*T*/298)^−(2.9±0.2)^ cm^3^ s^–1^.

The experimentally determined negative temperature dependence of *k*_1_ contrasts with an earlier theoretical prediction of a weak positive temperature dependence, which was impacted by the significant multi-reference character of the reaction. Qualitative calculations in this work using the Master Equation Solver for Multi-Energy well Reactions (MESMER) are able to reproduce a negative temperature dependence by reducing the calculated barrier heights for the reaction, but a significant discrepancy remains between measured and calculated rate coefficients. Further studies of product yields would be beneficial.

## Supporting Information Available

Analysis of absorbance spectra; concentration–time profiles for observed species; upper limit for yield of NO_3_; instrument response function; mixed-order fits; effects of pressure on *k*_1_ at 242 and 254 K; laser-induced fluorescence experiments to investigate potential production of NO; MESMER fits to experimental data for *k*_1_; investigation of potential indirect HCHO production; summary of experimental results; MESMER input file (PDF)
