# Supplementary material for: Kinetics of the Reaction Between the Criegee Intermediate CH2OO and NO2: Experimental Measurements and Comparison with Theory
Source: J Phys Chem A. 2025 Feb 12;129(8):2058–66. doi: 10.1021/acs.jpca.4c08203 (PMC11874013; doi:10.1021/acs.jpca.4c08203)
Supplement: Supplementary file 1 — jp4c08203_si_001.pdf [file jp4c08203_si_001.pdf]

Kinetics of the Reaction Between the Criegee Intermediate CH_2OO and NO_2: Experimental Measurements and Comparison with Theory

Rachel E. Lade[1], Kate A. Livesey[1], Luc Vereecken[2], Robin J. Shannon[1], Mark A. Blitz[1,3], Paul W. Seakins[1], and Daniel Stone[1*]

[1] School of Chemistry, University of Leeds, Leeds, LS2 9JT, UK

[2] Institute for Energy and Climate Research, ICE-3: Troposphere, Forschungszentrum Jülich GmbH, 52425, Jülich, Germany

[3] National Centre for Atmospheric Science, University of Leeds, Leeds, LS2 9JT, UK

*Corresponding author: Daniel Stone d.stone@leeds.ac.uk

Supporting Information

Contents

1. Analysis of absorbance spectra — Page S2

2. Concentration-time profiles for observed species — Page S5

3. Upper limit for yield of NO_3 — Page S6

4. Instrument response function — Page S8

5. Mixed-order fits — Page S9

6. Effects of pressure on k_1 at 242 and 254 K — Page S10

7. Laser-induced fluorescence experiments to investigate potential production of NO — Page S11

8. MESMER fits to experimental data for k_1 — Page S13

9. Investigation of potential indirect HCHO production — Page S15

10. Summary of experimental results — Page S17

11. References — Page S19

12. MESMER input file — Page S20

1. Analysis of absorbance spectra

Absorbance spectra obtained in the absence of NO_2 were observed to display characteristic absorption features of iodine monoxide (IO) radicals, as shown in Figure S1. IO generation has been reported in previous work, with IO produced from a combination of CH_2OO + I and CH_2IO_2 + I.[1]

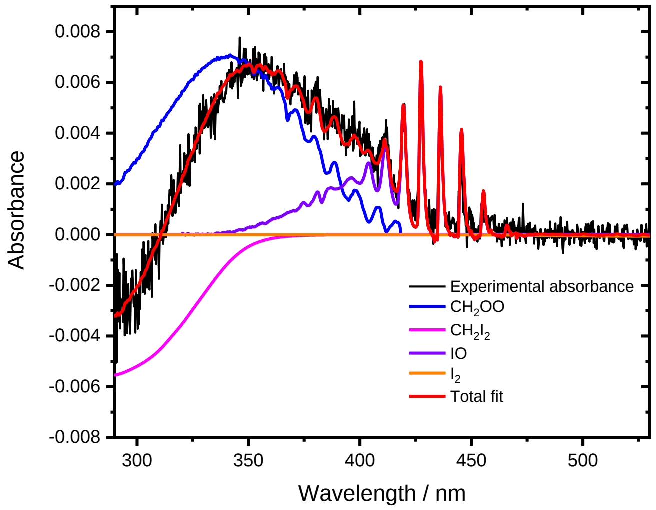

Figure S1: Typical absorbance spectrum obtained in the absence of NO_2 (black) with the total fit (red) and contributions to the total from CH_2I_2 (pink),[2] CH_2OO (blue),[1] IO (purple)[3] and I_2 (orange)[4] obtained by fitting reference spectra to the observations (Equation 1, main text). Data shown were obtained at 2 ms after photolysis at p = 100 Torr and T = 298 K, with $[CH_2I_2] = 5.9 \times 10^{13}$ cm^{-3} and $[O_2] = 4.0 \times 10^{17}$ cm^{-3}. The fit gave $\Delta[CH_2I_2]$ = 3.0×10^{12} cm^{-3}, $[CH_2OO] = 1.1 \times 10^{12}$ cm^{-3}, $[IO] = 6.7 \times 10^{11}$ cm^{-3} and $[I_2]$ = 0.

In the presence of NO_2, absorbance spectra displayed no, or little, production of IO radicals, but absorption consistent with production of INO_2 was observed, which was assumed to be produced via I + NO_2. Figure S2 shows fits to a typical absorbance spectrum obtained in the presence of NO_2, which includes the contribution from INO_2.

At wavelengths greater than 450 nm, absorption features consistent with the presence of I_2 were also observed, as shown in Figure S2, but it should be noted that additional absorbance in this region does not impact the determination of CH_2OO concentrations since there is no absorbance by CH_2OO in this region of the spectrum. The potential for production of $IONO_2$ was also investigated, but the inclusion of the reference spectrum for $IONO_2$ did not significantly improve the quality of the fits. Fits to spectra used to determine k_1 in this work did not include absorption cross-sections for $IONO_2$.

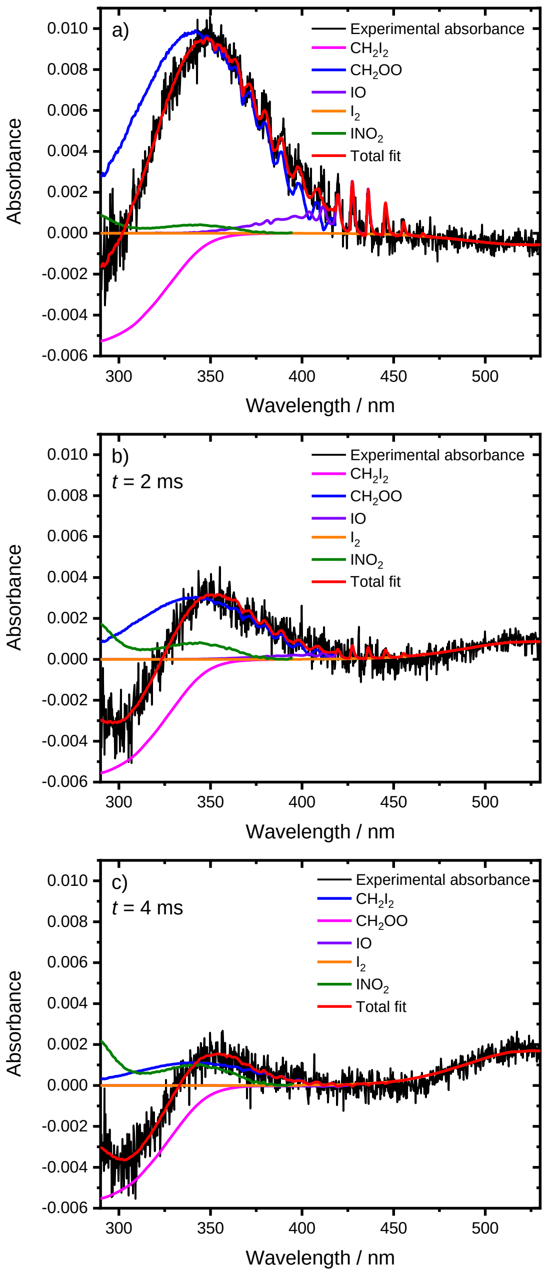

Figure S2: Typical observed absorbance (black) and total fit (red) obtained by fitting reference spectra for CH_2I_2 (pink),[2] CH_2OO (blue),[1] IO (purple),[3] I_2 (orange)[4] and INO_2 (green)[5] using Equation 1. Data shown were obtained at a) immediately following photolysis b) 2 ms and c) 4 ms after photolysis p = 100 Torr and T = 298 K, with $[CH_2I_2] = 5.9 \times 10^{13}$ cm^{-3}, $[O_2] = 4.0 \times 10^{17}$ cm^{-3}, and $[NO_2] = 8.1 \times 10^{14}$ cm^{-3}. The fits gave: a) $\Delta[CH_2I_2] = 2.9 \times 10^{12}$ cm^{-3}, $[CH_2OO] = 1.4 \times 10^{12}$ cm^{-3}, $[IO] = 1.2 \times 10^{11}$ cm^{-3}, $[I_2] = 0$ and $[INO_2] = 9 \times 10^{11}$ cm^{-3}; b) $\Delta[CH_2I_2] = 3.0 \times 10^{12}$ cm^{-3}, $[CH_2OO] = 4.7 \times 10^{11}$ cm^{-3}, $[IO] = 3.0 \times 10^{10}$ cm^{-3}, $[I_2] = 3.8 \times 10^{11}$ cm^{-3} and $[INO_2] = 2.4 \times 10^{12}$ cm^{-3}; c) $\Delta[CH_2I_2] = 3.1 \times 10^{12}$ cm^{-3}, $[CH_2OO] = 1.7 \times 10^{11}$ cm^{-3}, $[IO] = 1.3 \times 10^{10}$ cm^{-3}, $[I_2] = 4.2 \times 10^{12}$ cm^{-3} and $[INO_2] = 3.2 \times 10^{12}$ cm^{-3}.

Two sets of data for absorption cross-sections for INO_2 are available on the MPI Mainz UV/Vis Spectral Atlas, based on measurements obtained by Bröske[5] in which INO_2 was prepared by the photolysis of NO_2/I_2 mixtures using visible light at room temperature. However, Bröske reported significant uncertainties in the absorption cross-sections, and IUPAC recommend lower cross-sections which assume stoichiometric conversion of NO_2 into INO_2 in the work by Bröske. The two sets of data available on the MPI Mainz UV/Vis Spectral Atlas thus give the same shape of the spectrum, but differ in the magnitude of the absorption cross-sections, as shown in Figure S3.

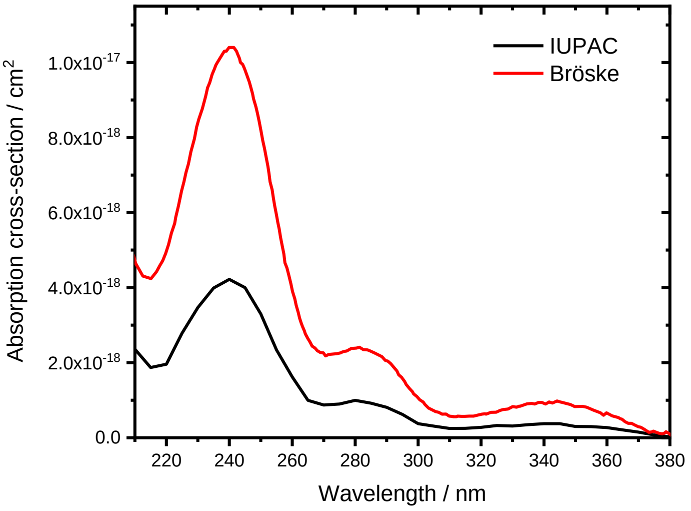

Figure S3: Absorption cross-sections for INO_2 available on the MPI Mainz UV/Vis Spectral Atlas reported by Bröske[5] (red) and IUPAC (black).[6]

Fits to data obtained in this work give significantly different concentrations of INO_2 depending on which absorption cross-sections were used in the fits, although it should be noted that concentrations of other species are not impacted. The total iodine concentration in the system can be estimated from the observed change in CH_2I_2 concentration on photolysis, and use of the IUPAC recommended cross-sections for INO_2 typically indicate concentrations of INO_2 that exceed the total available iodine concentration in the system whereas cross-sections reported by Bröske do not. Measurements in this work thus indicate that the cross-sections for INO_2 recommended by IUPAC, which are noted as being provisional, are too low. As a consequence, the observed absorbance spectra were fit using the INO_2 reference spectra reported by Bröske.[5]

2. Concentration-time profiles for observed species

Figure S4 gives typical concentration-time profiles for species observed in the system in the presence of NO_2, indicating a negative change in concentration of CH_2I_2 due to its depletion upon photolysis and the production of CH_2OO, IO, INO_2 and I_2 following photolysis.

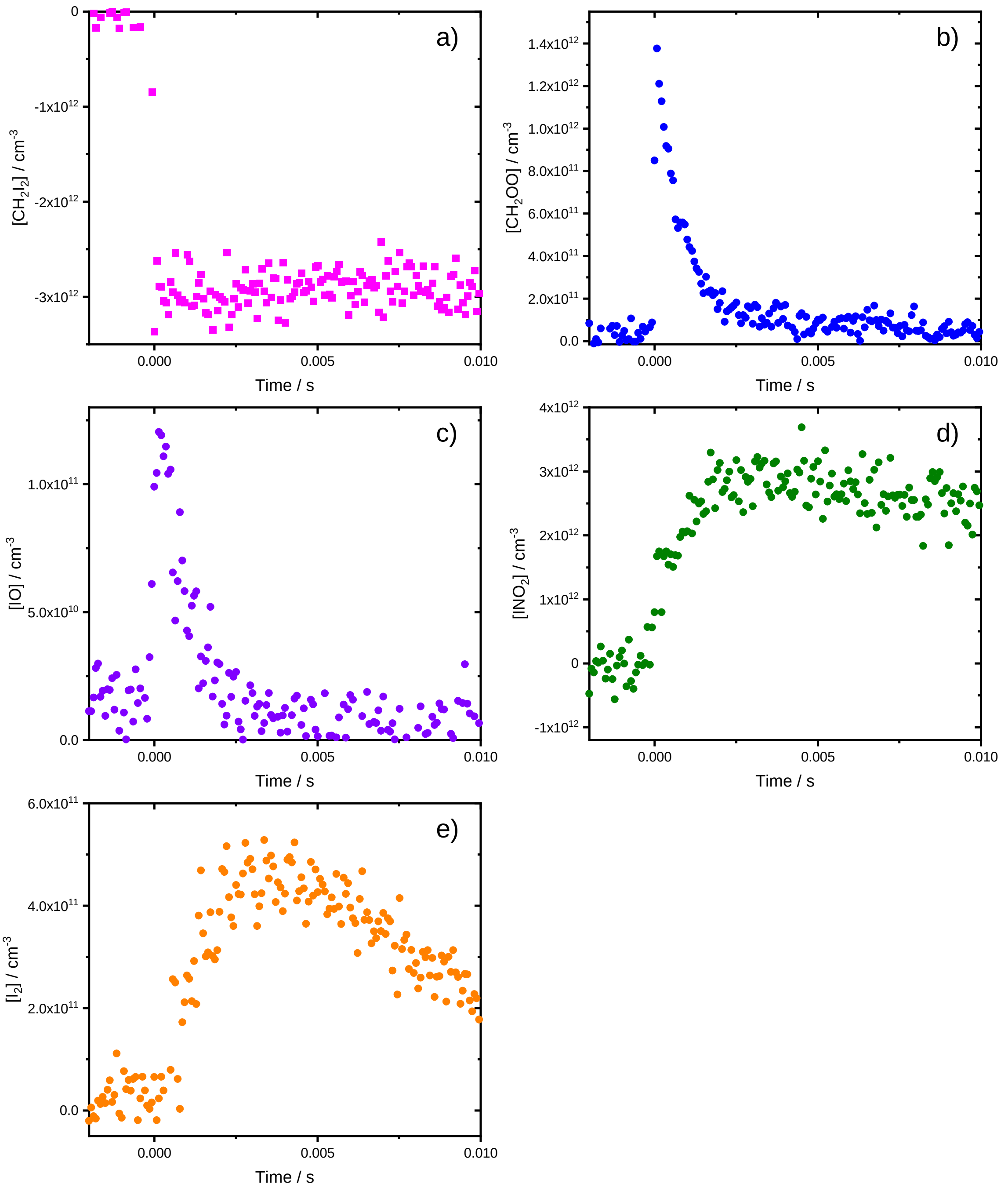

Figure S4. Typical concentration-time profiles for a) CH_2I_2 b) CH_2OO, c) IO, d) INO_2 and e) I_2 obtained at p = 100 Torr and T = 298 K, with $[CH_2I_2]$ = 5.9×10^{13} cm^{-3}, $[O_2]$ = 4.0×10^{17} cm^{-3} and $[NO_2]$ = 8.1×10^{14} cm^{-3}.

3. Upper limit for yield of NO_3

No significant evidence for production of NO_3 was observed in this work. Based on the standard deviation of the pre-photolysis absorbance, the effective pathlength of the probe beam, and the absorption cross-sections of NO_3 (which reach a maximum of ~2×10^{-17} cm^2 at $\lambda = 662$ nm), the limit of detection for NO_3 is estimated at 3×10^{10} cm^{-3}, which is ~3 % of the typical initial CH_2OO concentration.

On the timescale of experiments performed in this work, the reaction of NO_3 with NO_2 to produce N_2O_5 has the potential to impact the ability to observe NO_3. Simulations were performed using a simple kinetic model in which the production of NO_3 was treated as a pseudo-first-order reaction of CH_2OO with NO_2, using the rate coefficient obtained in this work at 298 K, with pseudo-first-order loss of NO_3 via $NO_3 + NO_2$ to produce N_2O_5 modelled using the IUPAC recommended rate coefficient at 298 K and 50 Torr.[6]

Figure S5 shows the impact of $NO_3 + NO_2$ on simulated NO_3 concentrations, indicating that any NO_3 produced from $CH_2OO + NO_2$ would be observable in the experiments reported in this work at a yield > 5 %. We therefore place an upper limit of < 5 % for the yield of NO_3 from the reaction of $CH_2OO + NO_2$.

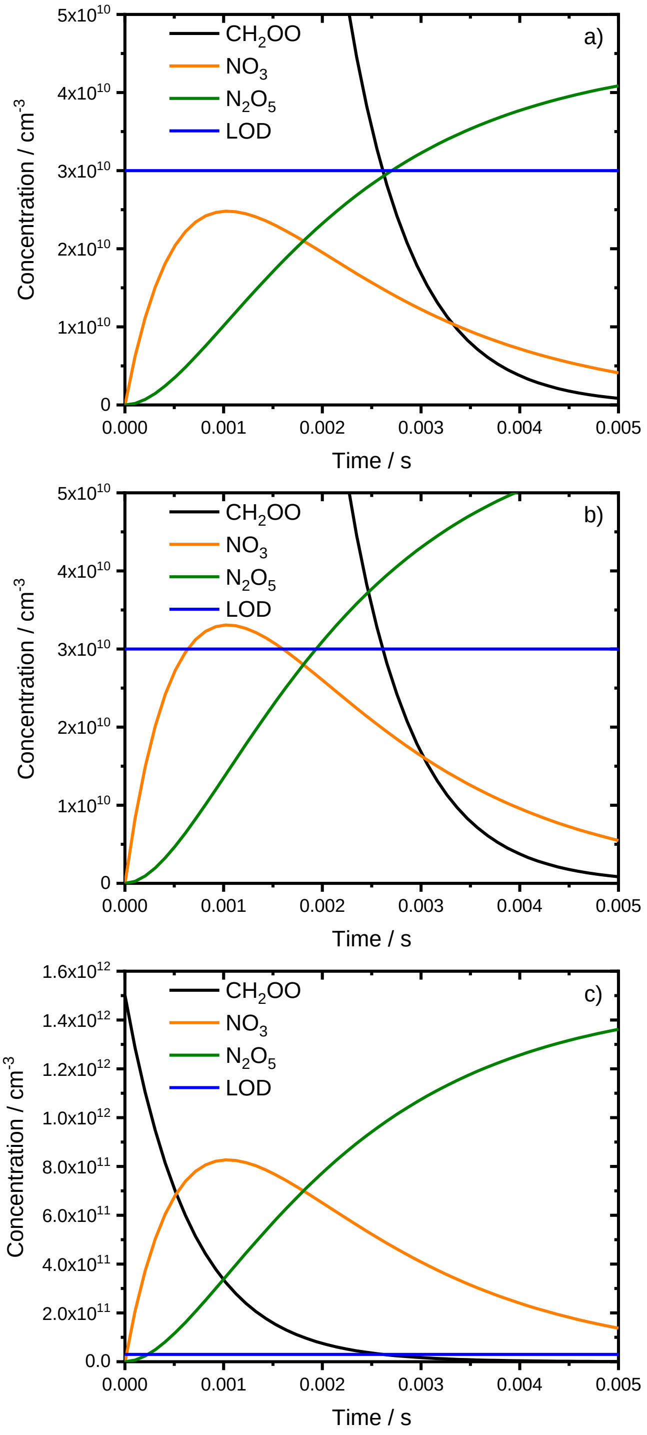

Figure S5: Simulated concentrations of CH2OO, NO_3, and N_2O_5 assuming production from CH_2OO + NO_2 and removal via NO_3 + NO_2 for a typical initial concentration of 1.5 × 10^{12} cm^{-3} CH_2OO in the presence of 10^{15} cm^{-3} NO_2 at 298 K and a total pressure of 50 Torr. Simulations are shown for NO_3 yields from CH_2OO + NO_2 of a) 3 %, b) 4 %, and c) 100 %. The estimated limit of detection of 3 × 10^{10} cm^{-3} for NO_3 is also shown (solid blue line), indicating that NO_3 would be observable at a yield > ~5 % in experiments performed in this work. The rate coefficient for CH_2OO + NO_2 was taken from results obtained in this work, while IUPAC recommendations[6] were used for NO_2 + NO_3.

4. Instrument Response Function

Concentration-time profiles observed in this work are given by a convolution of the 'true physical kinetic decay' kinetic decay with an instrument response function (IRF) which results from the simultaneous illumination of multiple rows on the charge-coupled device (CCD) detector (see main text for details) and the row-by-row shifting of photocharge from the illuminated region of the CCD to a storage region. The IRF can be described by a Gaussian function with peak height *a* centred at t_c and with width *w* (Equation S5):

$$f(t) = a\ exp\left(-\frac{(t-t_c)^2}{2w^2}\right)$$

(Equation S5)

The 'physical' kinetic decay for CH_2OO under pseudo-first-order conditions is given by Equation S6:

$$C_t = C_0\ exp(-k't)$$

(Equation S6)

where C_t is the concentration of CH_2OO at time *t*, C_0 is the initial concentration of CH_2OO, and *k'* is the pseudo-first-order rate coefficient describing the loss of the Criegee intermediate.

Convolution of the IRF (Equation S5) with the first-order (or pseudo-first-order) kinetic decay (Equation S6) gives Equation S7:

$$C_t = \frac{C_0}{2}\ exp\left\{\frac{(k'w)^2}{2} - k'(t-t_c)\right\} \times \left\{1 + \mathrm{erf}\left(\frac{t - t_c - k'w^2}{\sqrt{2}w}\right)\right\}$$

(Equation S7)

where erf is the error function obtained in the integration of the normalised form of the Gaussian function.

Results given in the main text were obtained by fitting Equation S7 to the observed concentration-time profiles for CH_2OO, with the IRF parameters t_c and *w* treated as global parameters.

The potential for mixed-first and second-order behaviour was also investigated (see below), in which case the 'true' kinetic decays are described by Equation S8:

$$C_t = \frac{C_0k'}{k'\ exp(k't) - 2k''C_0 + 2k''C_0\ exp(k't)}$$

(Equation S8)

where C_t is the concentration of CH_2OO at time *t*, C_0 is the initial concentration of CH_2OO, *k'* represents the first-order (or pseudo-first-order) losses of the Criegee intermediate and *k''* represents the second-order losses of Criegee intermediate.

Convolution of the IRF (Equation S5) with the mixed first- and second-order kinetic decay (Equation S8) gives Equation S9:

$$C_t = \left\{\frac{1}{\left(\frac{1}{C_0} + \frac{2k''}{k'}\right)}\right\} \exp\left\{\frac{(k'w)^2}{2} - k'(t - t_c) + \frac{2k''}{k'}\right\} \times \frac{\left\{1 + \mathrm{erf}\left(\frac{t - t_c - k'w^2}{\sqrt{2}\,w}\right)\right\}}{2}$$

(Equation S9)

Investigation of potential mixed-order behaviour described below was performed by fitting Equation S9 to the observed concentration-time profiles for CH_2OO, with the IRF parameters t_c and *w* treated as global parameters.

5. Mixed-order fits

Concentration-time profiles for CH_2OO were fit to both a first-order kinetic equation (Equation S6, coupled with the IRF to give Equation S7) and to a mixed first- and second-order kinetic equation (Equation S8, coupled with the IRF to give Equation S9) to investigate the potential impacts of any second-order reactions such as CH_2OO + CH_2OO or CH_2OO + I. For fits to the mixed-order equation, the second-order component (k'' in Equations S8 and S9) was treated as a global parameter at each temperature and pressure.

Figure S6 shows a comparison between the first-order and mixed-order fits to typical concentration-time profiles for CH_2OO at T = 298 K and p = 100 Torr, which indicates there were no significant differences between the first-order component obtained when describing the kinetics using a first-order model and when describing the kinetics using a mixed-order model.

Figure S7 compares rate coefficients for the reaction of CH_2OO with NO_2 obtained from the first-order fits to those obtained from the mixed-order fits. Results show only a 5 % difference between the rate coefficients obtained when kinetics were described using the first-order and mixed-order models and values obtained for the intercept, k_0, between the two fits within their error limits. We therefore conclude that data are well described by pseudo-first-order kinetics.

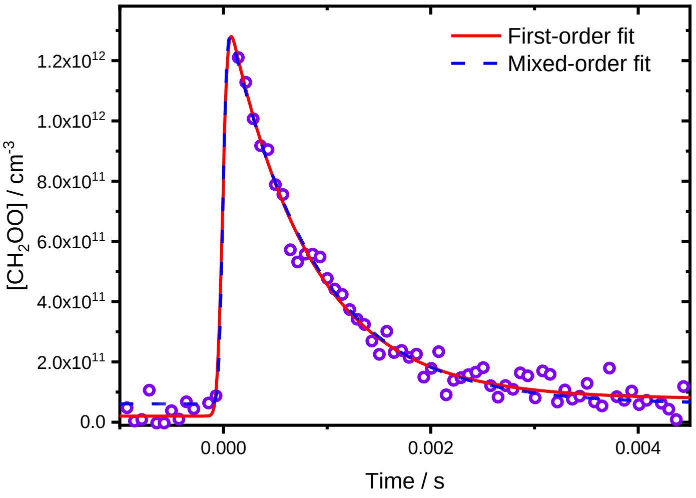

Figure S6. Comparison of fits to the first-order equation (Equation S7, solid red line) and the mixed-order equation (Equation S9, dashed blue line) for CH_2OO in the presence of $[NO_2] = 6.5 \times 10^{14}$ cm^{-3} at 100 Torr and 298 K. The solid red line gave: $t_c = -(1.05 \pm 0.13) \times 10^{-5}$ s, $w = (4.05 \pm 0.90) \times 10^{-5}$ s, $[CH_2OO]_0 = (1.37 \pm 0.24)\times 10^{12}$ cm^{-3} and $k' = (1271 \pm 44)$ s^{-1}. The dashed blue line gave: $t_c = -(1.18 \pm 0.58) \times 10^{-5}$ s, $w = (3.27 \pm 0.32) \times 10^{-5}$ s, $[CH_2OO]_0 = (1.39 \pm 0.29)\times 10^{12}$ cm^{-3}, $k'' = (9.99 \pm 0.25) \times 10^{11}$ cm^3 s^{-1} and $k' = (1198 \pm 49)$ s^{-1}. Uncertainties are 1σ.

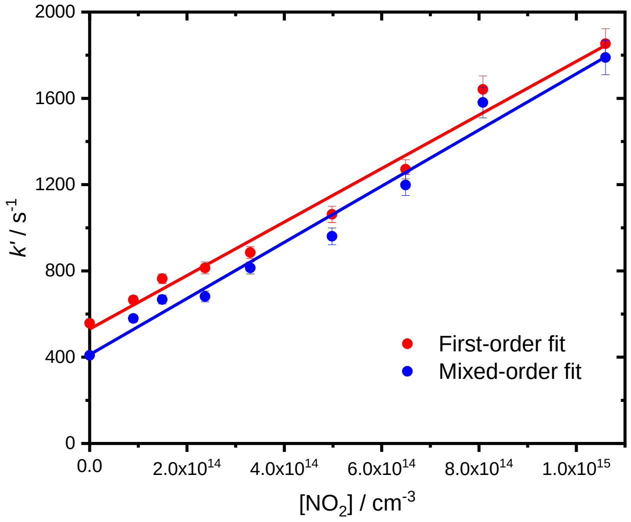

Figure S7. Dependence of k' on $[NO_2]$ at T = 298 K and p = 100 Torr obtained for a first-order fit (solid red line) and mixed-order fit (solid blue line). The fits gave: $k_1 = (1.24 \pm 0.07) \times 10^{-12}$ cm^3 s^{-1} and $k_0 = (531 \pm 35)$ s^{-1} for the first-order fit and $k_1 = (1.30 \pm 0.07) \times 10^{-12}$ cm^3 s^{-1} and $k_0 = (412 \pm 38)$ s^{-1} for the mixed-order fit. Uncertainties are 1σ.

6. Effects of pressure on k_1 at 242 and 254 K

Effects of pressure on k_1 were investigated at temperatures of 242 and 254 K, in addition to the experiments reported in the main text at 298 K. Figure S8 shows the results of experiments at 25, 50, and 200 Torr at 242 and 254 K, indicating that k_1 is not significantly affected by pressure under the conditions of experiments reported in this work.

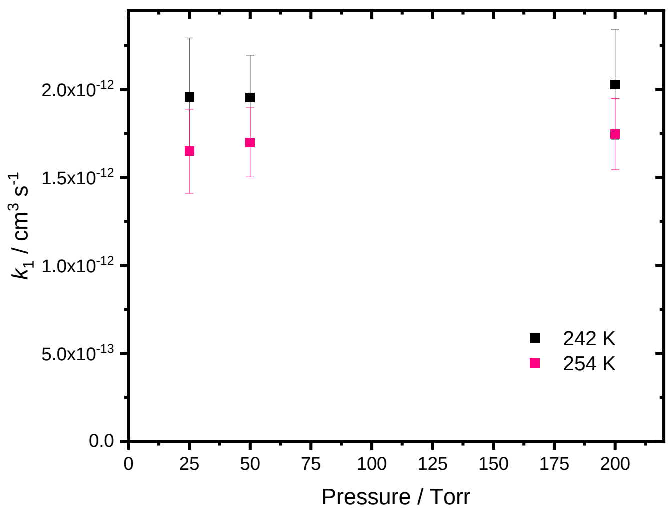

Figure S8. Bimolecular rate coefficients for the reaction between CH_2OO and NO_2 at pressures of 25 Torr, 50 Torr and 200 Torr for temperatures of 242 K (black) and 254 K (pink). At 242 K, results gave: $k_1 = (1.96 \pm 0.27)$ cm^3 s^{-1} at 25 Torr, $k_1 = (1.95 \pm 0.14)$ cm^3 s^{-1} at 50 Torr and $k_1 = (2.03 \pm 0.24)$ cm^3 s^{-1} at 200 Torr. At 254 K, results gave: $k_1 = (1.65 \pm 0.17)$ cm^3 s^{-1} at 25 Torr, $k_1 = (1.70 \pm 0.10)$ cm^3 s^{-1} at 50 Torr and $k_1 = (1.75 \pm 0.10)$ cm^3 s^{-1} at 200 Torr. Errors are 1σ.

7. Laser-induced fluorescence experiments to investigate potential production of NO

Experiments were performed using laser-induced fluorescence (LIF) spectroscopy to investigate the potential production of NO, which has been predicted to be a co-product of the channel producing HCHO (see Figure 1, main text). A series of preliminary experiments were carried out on a reaction that is known to produce NO to ensure that the system is capable of determining NO product kinetics with good sensitivity. The preliminary reaction studied was:

$$CH_3 + NO_2 \rightarrow CH_3O + NO \qquad \text{(RS1)}$$

This bimolecular reaction dominates at low pressure, but will be outcompeted at high-pressure by the association reaction, which forms CH_3NO_2. Reaction RS1 was initiated by the photolysis of CH_3I:

$$CH_3I + h\nu \text{ (248 nm)} \rightarrow CH_3 + I \qquad \text{(RS2)}$$

NO is a stable molecule, so to obtain kinetic information in these experiments requires the reaction cell to be largely replenished every laser pulse. This is arranged by using an increased gas flow and reducing the repetition rate of the lasers. After establishing the validity of the experiment, the gases were switched to the target reaction.

Experiments were performed using a slow-flow reactor that has been described in detail in previous work.[7, 8] Precursor gas mixtures, NO_2, CH_3I and CH_2I_2, were prepared in glass bulbs using a gas-line. These gases, together with the buffer gas (Ar) and oxygen were delivered through a mixing manifold using calibrated mass flow controllers and passed into the stainless steel reaction cell at typical flow rates of between 500 – 1000 sccm. The pressure was adjusted to be ~20 Torr to maintain a constant and short residence time in the cell of ~1 s. Pressure in the cell was monitored by a capacitance manometer (MKS Baratron, 0-1000 Torr) and controlled by a valve used to throttle the exit of the cell to a rotary pump. All experiments were performed at room temperature.

Chemistry in the cell was initiated by an excimer laser operating at 248 nm with a typical fluence of 30 mJ cm^{-2} and repetition rate 2 Hz. NO (v=0) was detected by LIF spectroscopy at ~226 nm using the doubled output of an Nd:YAG pumped (Continuum Precision II, 355 nm) dye laser (Sirah, Cobra-Stretch, energy < 5 mJ $pulse^{-1}$, Coumarin 440 dye) to excite close to the band-head in the A-X transition. Fluorescence from NO (v=0) at ~226 nm was detected by a photomultiplier (Electron Tubes) mounted perpendicular to the plane of the photolysis and probe beams. The photomultiplier signal was digitised, integrated by an oscilloscope (LeCroy LT 372), and passed to a computer for data analysis.

The time delay between the photolysis and probe lasers, controlled by a digital delay generator (BNC 555), was varied to enable monitoring as a function of time following photolysis. Typically, time traces consisted of 220 data points, each averaged 5-10 times.

The preliminary experiments monitored the growth of NO from the reaction of CH_3 + NO_2, which at low pressures produces CH_3O + NO (RS1). Production of NO was observed following photolysis of $CH_3I/NO_2/Ar/O_2$, as shown in Figure S9, with clear growth observed following the photolysis and little instant NO production from the photolysis of NO_2. Initial concentrations of CH_3 were ~10^{12} cm^{-3}, which is more than ten times the concentration of NO produced via direct photolysis of NO_2, which was present at concentrations up to ~10^{14} cm^{-3}. Analysis of the kinetics of NO growth were in agreement with expected kinetics of CH_3 + NO_2, indicating the capability to detect NO and to measure kinetics.

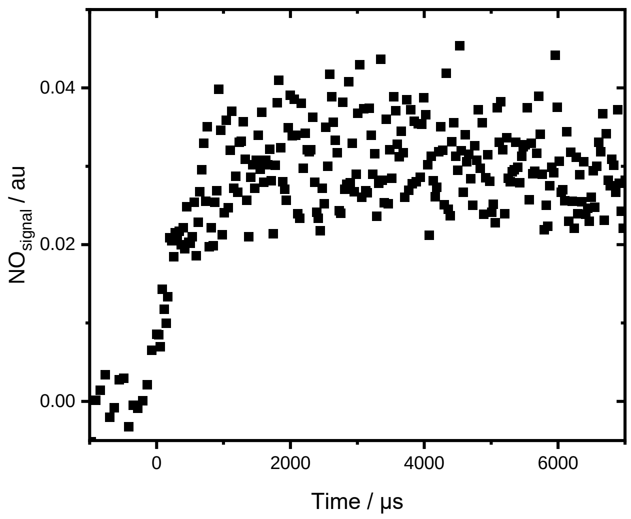

Figure S9. Typical growth NO trace determined from the reaction between CH_3 and NO_2.

Subsequent experiments were performed using photolysis of $CH_2I_2/O_2/NO_2/Ar$ gas mixtures to investigate the potential production of NO from the reaction of CH_2OO with NO_2. Figure S10 shows the NO signal obtained following photolysis of $CH_2I_2/O_2/NO_2/Ar$. While NO was observed, the signal was not significantly different from that observed following just photolysis of NO_2/Ar. This indicated production from photolysis of NO_2, but no significant production from $CH_2OO + NO_2$. While it is possible that any NO formed in the reaction between $CH_2OO + NO_2$ might be vibrationally excited, relaxation of NO (v=1) to NO (v=0) through collision with NO_2 has been demonstrated to have a rate coefficient of ~2 × 10^{-12} cm^3 s^{-1},[9] and vibrationally excited NO should be efficiently relaxed to the ground state, and thus observable, under our experimental conditions. We thus conclude that NO is not a significant product of the reaction between CH_2OO and NO_2, and place an upper limit of ~5 % on the yield of NO from $CH_2OO + NO_2$.

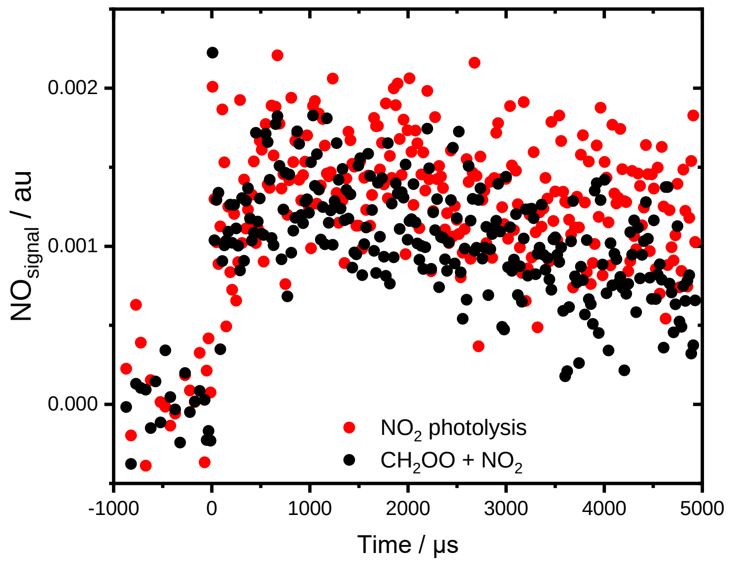

Figure S10. NO observed following the photolysis of NO_2/Ar (red) and NO observed following the photolysis of $CH_2I_2/O_2/NO_2/Ar$ (black). Results indicate that NO is only formed instantly from NO_2 photolysis and not from $CH_2OO + NO_2$. [CH_2I_2] = ~10^{14} cm^{-3}, [O_2] = 2 x 10^{17} cm^{-3}, [Ar] = 4 x 10^{17} cm^{-3}, [NO_2] = 2.5 x 10^{14} cm^{-3}.

8. MESMER fits to experimental data for k_1

MESMER fits described in the main text were performed in which the barrier height TS1 was varied, which gave a value for the energy of TS1 of -14 kJ mol^{-1}. Fits were also performed in which the barrier heights of both TS1 and TS2 were varied and in which only the barrier height of TS2 was varied, with the energy of TS1 fixed at the energy reported by Vereecken and Nguyen. For fits in which both TS1 and TS2 were varied the energy of TS1 needed to be lowered from -1 kJ mol^{-1} to -15 kJ mol^{-1} and that of TS2 needed to be lowered from 22 kJ mol^{-1} to -14 kJ mol^{-1}, with both fit values showing significant uncertainties (on the order of several hundred kJ mol^{-1}). The fitted value for the ILT parameter *A*, describing the formation of the pre-reaction complex, was (1.1 ± 3.3) × 10^{-12} cm^3 s^{-1}. For the fit in which only the energy of TS2 was varied (with the energy of TS1 fixed at -1 kJ mol^{-1}), the fitted value was -16 kJ mol^{-1}, with an uncertainty of < 10 kJ mol mol^{-1}, and *A* = (1.6 ± 1.9) × 10^{-12} cm^3 s^{-1}. In all fits, the ILT parameter *n* was fixed to its minimum value of -1.49 in order to best capture the observed temperature dependence. The fit quality was relatively insensitive to whether TS1 or TS2 or both TS1 and TS2 were varied, and no significant differences between fitted rate coefficients for the different fits were observed. The potential energy surfaces are summarised in Figure S11 and results are shown in Figure S12.

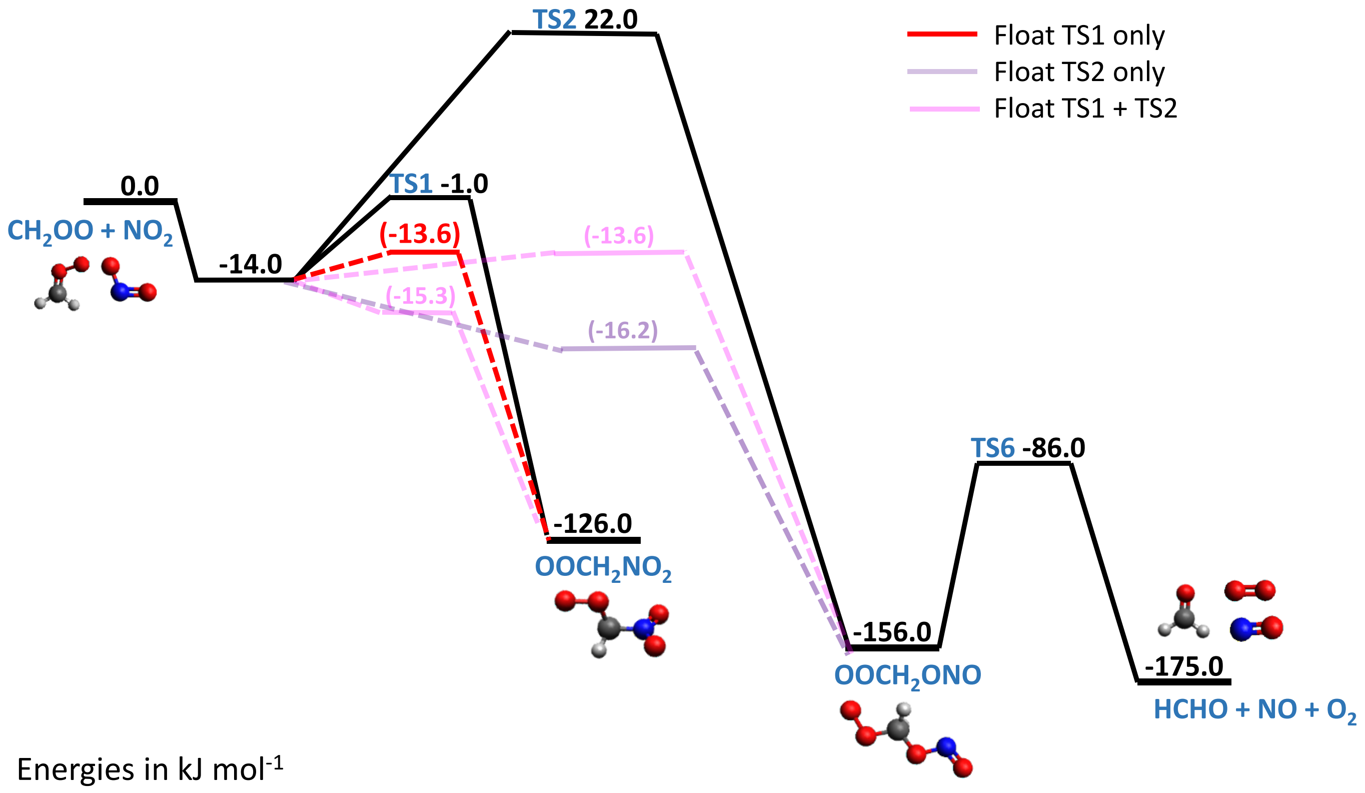

Figure S11: Simplified potential energy surface for the reaction between $CH_2OO + NO_2$ based on the results reported by Vereecken and Nguyen. Solid lines and values in black show the surface reported by Vereecken and Nguyen. Dashed lines and values in red show the result obtained by fitting the barrier height for TS1 to the experimental observations made in this work using MESMER. Dashed lines and values in pink show the result obtained by fitting the barrier height for TS1 and TS2, while those in purple show the result obtained by fitting only the barrier height for TS2.

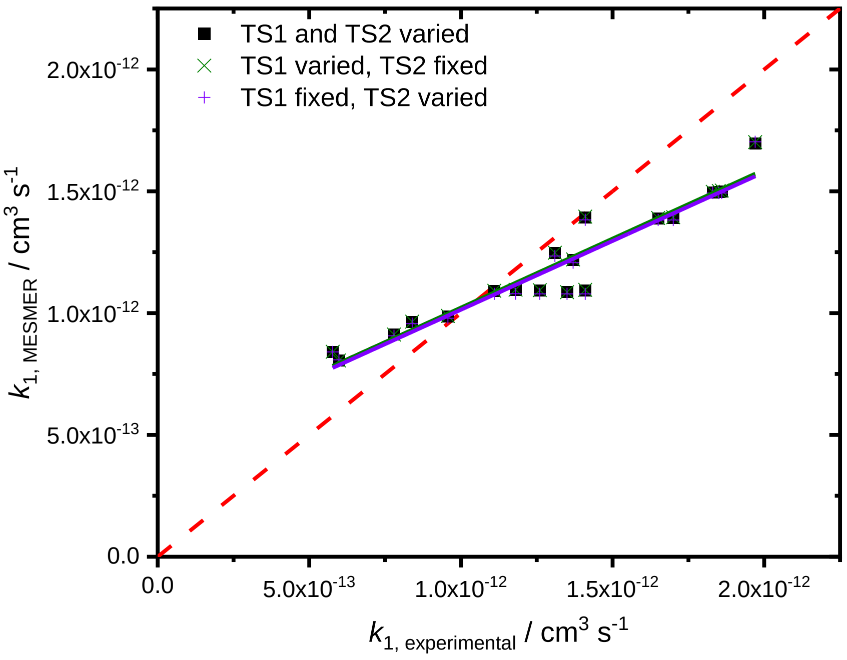

Figure S12: Comparison of MESMER fit results and observed values for k_1. Results are shown for fits in which a) TS1 and TS2 energies were varied (filled black squares) (see main text), b) TS1 was varied with TS2 fixed to 22 kJ mol^{-1} (green crosses), and c) TS2 was varied with TS1 fixed to -1 kJ mol^{-1} (purple plus signs). The best fits to the trends are given by a) $k_{1,\mathrm{MESMER}} = (0.56 \pm 0.04) \times k_{1,\mathrm{experimental}} + (4.58 \pm 0.56) \times 10^{-13}$ cm^3 s^{-1} ($r^2 = 0.92$), b) $k_{1,\mathrm{MESMER}} = (0.57 \pm 0.04) \times k_{1,\mathrm{experimental}} + (4.56 \pm 0.57) \times 10^{-13}$ cm^3 s^{-1} ($r^2 = 0.92$) and c) $k_{1,\mathrm{MESMER}} = (0.56 \pm 0.04) \times k_{1,\mathrm{experimental}} + (4.49 \pm 0.59) \times 10^{-13}$ cm^3 s^{-1} ($r^2 = 0.91$), and are shown by the solid lines. The 1:1 line is shown by the dashed red line.

9. Investigation of potential indirect HCHO production

In order to investigate the potential discrepancies in the nature of the products of CH_2OO + NO_2 between results reported in our earlier work[10] and observations made by Caravan *et al.*,[11] model calculations were performed to investigate the possible production of HCHO, observed in our earlier work, from the $O_2CH_2NO_2$ adduct observed by Caravan *et al*.

Production of the adduct was modelled as a function of NO_2 concentration using the mean rate coefficient determined in this work at 298 K (k_1 = (1.26 ± 0.11) × 10^{-12} cm^3 s^{-1}) and an initial CH_2OO concentration of 1 × 10^{12} cm^{-3}. Formation of HCHO from the $O_2CH_2NO_2$ adduct was modelled for a range of pseudo-first-order rate coefficients and the modelled HCHO was fit to a pseudo-first-order growth process and the result compared to the input $k_1[NO_2]$ used to model the production of the adduct.

Figure S13 shows the ratio of the fit result to the model input, with a ratio of unity indicating that HCHO formation from the $O_2CH_2NO_2$ adduct is sufficiently rapid to give the correct CH_2OO + NO_2 kinetics from observations of HCHO.[10] The model simulations indicate that conversion of an adduct between CH_2OO and NO_2 to HCHO with a first-order or pseudo-first-order rate coefficient on the order of ~10^3-10^4 s^{-1} could explain the observations made in our earlier work that CH_2OO is titrated to HCHO with ~100 % yield, whilst also providing the potential for observation of the adduct by the sensitive PIMS technique. Vereecken and Nguyen[12] suggested that the adduct $O_2CH_2NO_2$ would be stable with respect to unimolecular decomposition, but bimolecular reactions with other species in the system are possible, and expected to display similarities with reactions of peroxy radicals. The $O_2CH_2NO_2$ adduct is thus expected to react with species such as NO to produce OCH_2NO_2, which is expected to decompose rapidly to produce HCHO and NO_2.[12] It is possible that NO was produced in sufficient concentration in our earlier experiments,[10] through photolysis of NO_2 by the photolysis laser or by the LIF probe laser, to titrate the $O_2CH_2NO_2$ adduct to HCHO. Production involving the LIF probe laser in particular would lead to higher NO concentrations in our previous work compared to the experiments performed by Caravan *et al.*,[11] and thus more significant conversion of the adduct to HCHO. Direct photolysis of the $O_2CH_2NO_2$ adduct by the LIF probe laser to produce HCHO in our previous work is also a possibility, and would lead to rapid conversion of the adduct to HCHO. Further experiments to investigate product yields would be beneficial.

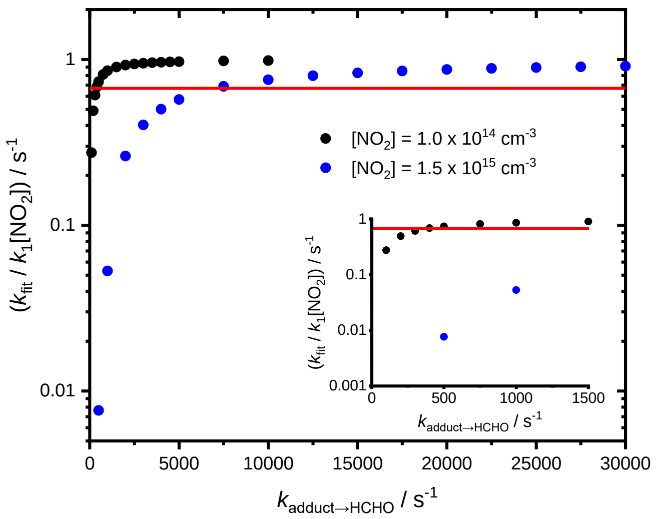

Figure S13: Ratio of the fit result for the kinetics of HCHO production from $O_2CH_2NO_2$ to the model input for CH_2OO + NO_2 for NO_2 concentrations of 1 × 10^{14} cm^{-3} (black) and 1.5 × 10^{15} cm^{-3} (blue). The red line represents a ratio of 0.67 (i.e. unity within the experimental uncertainty of our previous measurements of k_1), which indicates the point where HCHO formation from the adduct is sufficiently rapid to give the correct CH_2OO + NO_2 kinetics from observations of HCHO within the uncertainty of the measurements.

Further experiments were performed in which the kinetics of HCHO production following photolysis of $CH_2I_2/O_2/NO_2$ mixtures at λ = 266 nm and λ = 355 nm were monitored via LIF at ~353.1 nm at a total pressure of 20 Torr and temperature of 298 K. Compared to our previous experiments in which HCHO was monitored via LIF using a probe wavelength of ~353.1 nm following photolysis at λ = 248 nm,[10] experiments in this work were performed with higher total flows through the reaction cell and using O_2 as the bath gas rather than N_2, such that any NO formed in the current work was more rapidly flushed out of the reaction cell and/or converted to NO_2 compared to our earlier work. Assuming production of HCHO by $CH_2OO + NO_2$, experiments in which the chemistry was initiated at λ = 355 nm gave a rate coefficient for $CH_2OO + NO_2$ of $(2.3 \pm 0.3) \times 10^{-12}$ cm^3 s^{-1}, in keeping with results obtained from direct observations of CH_2OO made by UV absorption spectroscopy in this work and by HCHO LIF measurements in our earlier work. However, results obtained following photolysis at λ = 266 nm gave a rate coefficient of $(1.9 \pm 0.5) \times 10^{-13}$ cm^3 s^{-1}, with the difference attributed to the significant reduction in production of NO from photolysis of NO_2 at 266 nm compared to 355 nm and the more rapid removal of any NO formed from the reaction cell compared to our earlier work.

Results are shown in Figure S14 and indicate that HCHO is formed indirectly in the system, likely following initial production of the $O_2CH_2NO_2$ adduct from $CH_2OO + NO_2$ followed by rapid reaction of the adduct with NO to produce OCH_2NO_2 and NO_2 and decomposition of OCH_2NO_2 to produce the HCHO observed and NO_2.

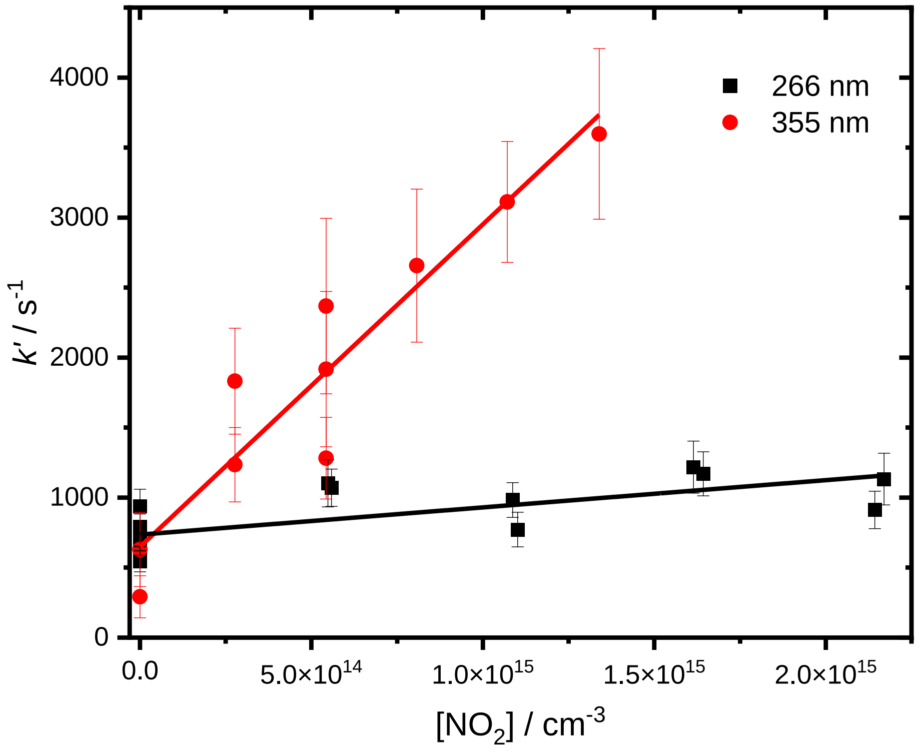

Figure S14: Dependence of the pseudo-first-order rate coefficients describing the production of HCHO following photolysis of $CH_2I_2/O_2/N_2/NO_2$ mixtures at λ = 266 nm (black data points) and λ = 355 nm (red data points) on the concentration of NO_2. Assuming production of HCHO by $CH_2OO + NO_2$, experiments in which the chemistry was initiated at λ = 266 nm gave a rate coefficient for $CH_2OO + NO_2$ of $(1.9 \pm 0.5) \times 10^{-13}$ cm^3 s^{-1}, while experiments in which the chemistry was initiated at λ = 355 nm gave a rate coefficient for $CH_2OO + NO_2$ of $(2.3 \pm 0.3) \times 10^{-12}$ cm^3 s^{-1}.

10. Summary of experimental results

Temperature / K	Pressure / Torr	$[CH_2I_2]$ / 10^{13} cm^{-3}	$[NO_2]$ / 10^{14} cm^{-3}	k' / s^{-1}
**242**	25	5.3	0	761 ± 29
			1.9	1503 ± 78
			4.7	2026 ± 116
			5.7	2020 ± 126
			7.3	2620 ± 200
			9.8	2692 ± 239
**242**	50	6.1	0	761 ± 19
			2.6	1417 ± 89
			3.5	1512 ± 95
			4.8	1561 ± 91
			6.2	1965 ± 122
			7.3	2225 ± 172
			8.1	2431 ± 181
**242**	200	7.1	0	461 ± 21
			1.5	1000 ± 102
			3.6	1574 ± 202
			6.7	1747 ± 299
			10.8	2837 ± 429
**254**	25	5.4	3.3	1018 ± 35
			4.6	1452 ± 65
			5.3	1682 ± 81
			7.8	1804 ± 95
			8.2	2146 ± 142
			9.7	2261 ± 150
			12.9	2720 ± 187
**254**	50	4.6	4.7	977 ± 29
			5.9	1202 ± 39
			7.2	1489 ± 55
			8.3	1616 ± 61
			9.3	1836 ± 75
			10.4	1923 ± 85
**254**	200	5.6	0	573 ± 20
			1.8	1040 ± 56
			2.7	1024 ± 76
			3.9	1373 ± 85
			5.2	1490 ± 113
			7.0	1801 ± 140
			7.9	2041 ± 180
**277**	50	4.0	0	603 ± 14
			1.1	807 ± 21
			1.7	860 ± 28
			2.8	896 ± 33
			4.8	1175 ± 43
			6.9	1321 ± 54

			8.7	1843 ± 82
			10.4	2294 ± 122
			12.1	1961 ± 80
**298**	25	6.1	0	608 ± 11
			4.5	985 ± 22
			6.1	1116 ± 27
			10.6	1740 ± 53
			12.3	2022 ± 71
			14.3	2242 ± 86
**298**	50	5.9	5.0	945 ± 27
			5.8	970 ± 28
			6.7	1063 ± 30
			8.4	1197 ± 39
			9.7	1397 ± 47
			10.5	1405 ± 49
			13.3	1887 ± 66
**298**	100	5.5	0	557 ± 13
			0.9	666 ± 18
			1.5	764 ± 22
			2.4	814 ± 28
			3.3	885 ± 27
			5.0	1062 ± 38
			6.5	1271 ± 44
			8.1	1642 ± 62
			10.6	1853 ± 70
**298**	200	6.3	0	579 ± 14
			8.8	695 ± 27
			2.2	875 ± 45
			4.7	1169 ± 68
			6.4	1410 ± 81
			8.0	1725 ± 95
**298**	300	6.1	0	574 ± 16
			2.1	844 ± 39
			2.9	1069 ± 45
			4.5	1270 ± 65
			6.1	1393 ± 68
			9.8	1836 ± 85
**318**	50	4.8	0	564 ± 15
			2.4	715 ± 24
			4.3	982 ± 35
			6.0	1006 ± 37
			7.6	1325 ± 57
			8.5	1310 ± 54
			10.2	1690 ± 93
			13.1	1657 ± 62
**335**	50	7.5	0	653 ± 12
			1.1	763 ± 15
			1.6	786 ± 20

			3.2	827 ± 28
			5.0	955 ± 26
			6.5	1126 ± 31
			8.9	1380 ± 51
**353**	50	5.7	0	488 ± 13
			1.3	621 ± 16
			3.3	744 ± 20
			4.3	786 ± 20
			5.2	779 ± 27
			6.7	908 ± 30

Table S2. Experimental data obtained for experiments to investigate the kinetics of CH_2OO and NO_2 between 25 and 200 Torr and 242 and 353 K.

11. References

1. Mir, Z.S., Lewis, T.R., Onel, L., Blitz, M.A., Seakins, P.W. and Stone, D. CH_2OO Criegee intermediate UV absorption cross-sections and kinetics of CH_2OO + CH_2OO and CH_2OO + I as a function of pressure. *Physical Chemistry Chemical Physics.* 2020, **22**(17), pp.9448-9459.
2. Atkinson, R., Baulch, D.L., Cox, R.A., Crowley, J.N., Hampson, R.F., Hynes, R.G., Jenkin, M.E., Rossi, M.J., Troe, J. and Wallington, T.J. Evaluated kinetic and photochemical data for atmospheric chemistry: Volume IV – gas phase reactions of organic halogen species. *Atmos. Chem. Phys.* 2008, **8**(15), pp.4141-4496.
3. Harwood, M.H., Burkholder, J.B., Hunter, M., Fox, R.W. and Ravishankara, A.R. Absorption Cross Sections and Self-Reaction Kinetics of the IO Radical. *The Journal of Physical Chemistry A.* 1997, **101**(5), pp.853-863.
4. Atkinson, R., Baulch, D.L., Cox, R.A., Crowley, J.N., Hampson, R.F., Hynes, R.G., Jenkin, M.E., Rossi, M.J. and Troe, J. Evaluated kinetic and photochemical data for atmospheric chemistry: Volume III - gas phase reactions of inorganic halogens. *Atmospheric Chemistry and Physics.* 2007, **7**(4), pp.981-1191.
5. Bröske, R. *Kinetische und spektroskopische Untersuchungen von Nitrylhalogeniden, Halogennitriten und Halogennitraten*. thesis, University of Wuppertal, Germany, 2000.
6. Atkinson, R., Baulch, D.L., Cox, R.A., Crowley, J.N., Hampson, R.F., Hynes, R.G., Jenkin, M.E., Rossi, M.J. and Troe, J. Evaluated kinetic and photochemical data for atmospheric chemistry: Volume I - gas phase reactions of Ox, HOx, NOx and SOx species. *Atmospheric Chemistry and Physics.* 2004, **4**(6), pp.1461-1738.
7. Blitz, M.A., Onel, L., Robertson, S.H. and Seakins, P.W. Studies on the Kinetics of the CH + H_2 Reaction and Implications for the Reverse Reaction, $^3CH_2$ + H. *The Journal of Physical Chemistry A.* 2023, **127**(10), pp.2367-2375.
8. Potter, D.G., Wiseman, S., Blitz, M.A. and Seakins, P.W. Laser Photolysis Kinetic Study of OH Radical Reactions with Methyl *tert*-Butyl Ether and Trimethyl Orthoformate under Conditions Relevant to Low Temperature Combustion: Measurements of Rate Coefficients and OH Recycling. *The Journal of Physical Chemistry A.* 2018, **122**(50), pp.9701-9711.
9. Hancock, G., Morrison, M. and Saunders, M. Vibrational relaxation of NO (v = 1–16) with NO, N_2O, NO_2, He and Ar studied by time-resolved Fourier transform infrared emission. *Physical Chemistry Chemical Physics.* 2009, **11**(38), pp.8507-8515.
10. Stone, D., Blitz, M., Daubney, L., Howes, N.U.M. and Seakins, P. Kinetics of CH_2OO reactions with SO_2, NO_2, NO, H_2O and CH_3CHO as a function of pressure. *Physical Chemistry Chemical Physics.* 2014, **16**(3), pp.1139-1149.
11. Caravan, R.L., Khan, M.A.H., Rotavera, B., Papajak, E., Antonov, I.O., Chen, M.-W., Au, K., Chao, W., Osborn, D.L., Lin, J.J.-M., Percival, C.J., Shallcross, D.E. and Taatjes, C.A. Products of Criegee intermediate reactions with NO_2: experimental measurements and tropospheric implications. *Faraday Discussions.* 2017, **200**(0), pp.313-330.
12. Vereecken, L. and Nguyen, H.M.T. Theoretical Study of the Reaction of Carbonyl Oxide with Nitrogen Dioxide: CH_2OO + NO_2. *International Journal of Chemical Kinetics.* 2017, **49**(10), pp.752-760.

12. MESMER input file


```
<?xml version="1.0" encoding="utf-8" ?>
<?xml-stylesheet type='text/xsl' href='../../mesmer2.xsl' media='other'?>
<?xml-stylesheet type='text/xsl' href='../../mesmer1.xsl' media='screen'?>
<me:mesmer xmlns="http://www.xml-cml.org/schema" xmlns:me="http://www.chem.leeds.ac.uk/mesmer"
xmlns:xsi="http://www.w3.org/2001/XMLSchema-instance" xmlns:cml="http://www.xml-cml.org/schema">

<me:title>CH2OO + NO2</me:title>
<moleculeList>

 <molecule id="CH2OO">
   <propertyList>
   <property title="Energy" dictRef="me:ZPE">
   <scalar units="kJ/mol">0</scalar>
   </property>
   <property title="Vibrational Frequencies" dictRef="me:vibFreqs">
    <array units="cm-1"> 537.7503 697.5723 1022.3957 1261.0787 1630.8711 3141.7356</array>
   </property>
   <property title="Rotational Constants" dictRef="me:rotConsts">
    <array units="GHz"> 81.19793 12.6778 10.96568 </array>
   </property>
  <property dictRef="me:frequenciesScaleFactor">
   <scalar>1</scalar>
  </property>
  <property dictRef="me:symmetryNumber">
   <scalar>1</scalar>
  </property>
  <property dictRef="me:MW">
   <scalar units="amu">46</scalar>
  </property>
```

```
 &lt;property dictRef="me:spinMultiplicity"&gt;
  &lt;scalar&gt;1&lt;/scalar&gt;
 &lt;/property&gt;
 &lt;property dictRef="me:symmetryNumber"&gt;
  &lt;scalar&gt;2&lt;/scalar&gt;
 &lt;/property&gt;
&lt;/propertyList&gt;
&lt;me:DOSCMethod name="ClassicalRotors"/&gt;
&lt;me:energyTransferModel xsi:type="me:ExponentialDown"&gt;
&lt;me:deltaEDown units="cm-1"&gt;250&lt;/me:deltaEDown&gt;
&lt;/me:energyTransferModel&gt;
   &lt;/molecule&gt;


 &lt;molecule id="NO2"&gt;
  &lt;propertyList&gt;
  &lt;property title="Energy" dictRef="me:ZPE"&gt;
  &lt;scalar units="kJ/mol"&gt;0&lt;/scalar&gt;
  &lt;/property&gt;
  &lt;property title="Vibrational Frequencies" dictRef="me:vibFreqs"&gt;
   &lt;array units="cm-1"&gt; 783.454 1465.3073 1775.7005&lt;/array&gt;
  &lt;/property&gt;
  &lt;property title="Rotational Constants" dictRef="me:rotConsts"&gt;
   &lt;array units="GHz"&gt;254.03982 13.29059 12.62984&lt;/array&gt;
  &lt;/property&gt;
 &lt;property dictRef="me:frequenciesScaleFactor"&gt;
  &lt;scalar&gt;1&lt;/scalar&gt;
 &lt;/property&gt;
 &lt;property dictRef="me:symmetryNumber"&gt;
  &lt;scalar&gt;2&lt;/scalar&gt;
 &lt;/property&gt;
```

```
  <property dictRef="me:MW">
   <scalar units="amu">46</scalar>
  </property>
  <property dictRef="me:spinMultiplicity">
   <scalar>2</scalar>
  </property>
 </propertyList>
 <me:DOSCMethod name="ClassicalRotors"/>
 <me:energyTransferModel xsi:type="me:ExponentialDown">
 <me:deltaEDown units="cm-1">250</me:deltaEDown>
 </me:energyTransferModel>
  </molecule>

  <molecule id="prc">
   <propertyList>
   <property title="Energy" dictRef="me:ZPE">
   <scalar units="kJ/mol">-14</scalar>
   </property>
   <property title="Vibrational Frequencies" dictRef="me:vibFreqs">
        <array units="cm-1">36.5724 107.5697 110.1268 162.9850 194.5654 210.5406 540.5284 699.1192 781.9472 920.7260 1053.4548 1261.2692 1439.7321 1450.9565 1642.9652 1768.4335 3142.5373 3290.3128</array>
   </property>
   <property title="Rotational Constants" dictRef="me:rotConsts">
    <array units="GHz">7.45177 2.19286 1.79541</array>
   </property>
  <property dictRef="me:frequenciesScaleFactor">
   <scalar>1</scalar>
  </property>
  <property dictRef="me:symmetryNumber">
   <scalar>2</scalar>
  </property>
```

```
  <property dictRef="me:MW">
   <scalar units="amu">92</scalar>
  </property>
  <property dictRef="me:spinMultiplicity">
   <scalar>2</scalar>
  </property>
 </propertyList>
 <me:DOSCMethod name="ClassicalRotors"/>
 <me:energyTransferModel xsi:type="me:ExponentialDown">
 <me:deltaEDown units="cm-1">250</me:deltaEDown>
 </me:energyTransferModel>
  </molecule>

<molecule xmlns="http://www.xml-cml.org/schema" spinMultiplicity="2" id="TS1">
    <atomArray>
    <atom id="a1" elementType="C" hydrogenCount="2" x3="-1.003917" y3="1.152438" z3="-0.189718"/>
    <atom id="a2" elementType="N" hydrogenCount="0" x3="1.242808" y3="-0.023190" z3="-0.079844"/>
    <atom id="a3" elementType="O" hydrogenCount="0" x3="-1.615194 " y3="0.058976" z3="-0.391825"/>
    <atom id="a4" elementType="H" hydrogenCount="0" x3="-0.733346" y3="1.418446" z3="0.827089"/>
    <atom id="a5" elementType="H" hydrogenCount="0" x3="-0.892113" y3="1.787891" z3="-1.059485"/>
    <atom id="a6" elementType="O" hydrogenCount="0" x3="2.125785" y3="0.598784" z3="0.428986"/>
    <atom id="a7" elementType="O" hydrogenCount="0" x3="1.127361" y3="-1.146678" z3="-0.442632"/>
    <atom id="a8" elementType="O" hydrogenCount="0" x3="-1.769288" y3="-0.755912" z3="0.646671"/>
    </atomArray>
    <bondArray>
    <bond atomRefs2="a5 a1" order="1"/>
    <bond atomRefs2="a7 a2" order="1"/>
    <bond atomRefs2="a3 a1" order="1" id="b2"/>
    <bond atomRefs2="a3 a8" order="1"/>
    <bond atomRefs2="a1 a4" order="1"/>
    <bond atomRefs2="a2 a6" order="2"/>
```

<bond atomRefs2="a1 a2" order="1" id="b1"/>

</bondArray>

<propertyList>

<property dictRef="me:ZPE">

<!-- <scalar units="kJ/mol">-1</scalar> -->

<scalar units="kJ/mol" upper="30" lower="-20" stepsize="0.1" >-13.6</scalar>

</property>

<property dictRef="me:spinMultiplicity">

<scalar units="cm-1">2</scalar>

</property>

<property dictRef="me:hessian">

<matrix matrixType="squareSymmetricLT" rows="24" units="Hartree/Bohr2">0.15381251 0.21562422 0.61189263 0.05319560 -0.07616602 0.73371541 0.04238448 0.01528405 0.01818776 0.51820900 -0.03855624 -0.01838975 -0.01167084 0.22025323 0.82974498 0.00240617 0.00118233 -0.00470393 0.26654700 0.33312803 0.22556779 -0.08462447 -0.14382306 -0.04588697 -0.03438209 0.02600126 -0.00150979 0.15071756 -0.08228321 -0.28765038 -0.04849821 -0.03845621 0.04307148 0.00054316 0.16891633 0.41466328 -0.05749329 -0.07664634 -0.15516902 -0.00738563 -0.00400902 -0.00224592 0.05154372 -0.04511899 0.38744641 -0.04792106 -0.01637439 -0.05855182 -0.00552243 0.00675368 0.00369509 0.00309886 -0.02912857 -0.02331122 0.04958694 -0.02744105 -0.07098306 -0.04775631 0.00491096 -0.00366241 -0.00151234 -0.01677909 -0.00395199 -0.02967260 0.03080040 0.06582182 -0.06648500 -0.05541819 -0.33185310 -0.00091386 0.00023480 0.00073267 -0.00964460 -0.00968773 -0.00057514 0.07728046 0.06748773 0.33823662 -0.03248895 -0.02351019 0.01736121 -0.00089379 0.00229957 -0.00125032 -0.01180781 -0.01900625 0.02085896 0.00522730 0.00379304 -0.00418339 0.03266137 -0.03050764 -0.14774954 0.13639283 0.00003869 0.00004651 0.00052787 -0.00535095 -0.02949153 0.02795390 0.00046921 0.00915339 -0.00639787 0.03966780 0.16572648 0.02105537 0.14592870 -0.26375159 0.00045365 -0.00140539 0.00062495 0.00343685 -0.00169681 0.01054542 0.00391475 0.01314566 -0.00524399 -0.02659280 -0.15392516 0.25344993 -0.01071746 -0.00590222 -0.00712006 -0.44060181 -0.18742073 -0.23106705 0.00398965 0.00935635 0.00233797 0.00032867 -0.00055222 0.00047556 -0.00021681 0.00043321 0.00008956 0.44373364 0.00756744 0.00445099 0.00121590 -0.19433642 -0.19132726 -0.13403607 -0.00509915 -0.00933328 0.00241055 -0.00129497 0.00055297 0.00026834 -0.00065530 -0.00000762 0.00031342 0.27285079 0.27270427 0.00048117 -0.00054084 0.00028237 -0.23565928 -0.13041227 -0.14160159 -0.00136029 -0.00168300 0.00142526 -0.00109175 0.00038981 -0.00012480 0.00005576 0.00014757 -0.00003300 0.26065639 0.19168240 0.16656399 -0.00654699 -0.00150895 -0.00186148 -0.08538346 -0.02553564 -0.03886937 0.00371822 0.00767685 0.00017379 0.00000769 -0.00019599 0.00007175 0.00004550 0.00006179 0.00012963 0.00505978 -0.07968902 -0.02275193 0.08427215 0.01425925 0.00353956 0.00315676 -0.01693593 -0.64324305 -0.19716993 -0.01091980 -0.01760997 0.00475047 -0.00092808 0.00025781 -0.00021240 -0.00032159 -0.00063446 0.00026928 -0.08727189 -0.08120220 -0.06106087 0.10088066 0.73066783 -0.00060020 -0.00062141 0.00176126 -0.03221923 -0.20082689 -0.07978250 -0.00034909 -0.00022408 0.00109803 -0.00043229 0.00000319 -0.00027395 0.00010808 -0.00021019 -0.00028021 -0.02797783 -0.05808858 -0.02593340 0.06106959 0.25882757 0.10487495 -0.01389806 -0.03978945 0.02467576 0.00619010 -0.00379513 0.00004828 -0.03070992 -0.01707529 0.01327570 -0.00480597 0.00546395 0.00339906 0.00747318 -0.00481210 -0.00248700 -0.00157566 0.00065663 -0.00033006 -0.00117290 0.00123737 0.00040096 0.03849924 -0.05866278 -0.09511044 0.04332590 0.00924163 -0.01624050 -0.00266305 -0.01294554 -0.10969760 0.12033204 0.00970271 0.00281146 0.00372531 -0.00226707 0.00295677 -0.00262969 -0.00149328 0.00416212 0.00147720 -0.00168969 0.00822448 0.00114039 0.05811403 0.20289372 0.04744019 0.06228177 0.01971860 -0.00901040 0.01496159 0.00140854 0.00377016 0.10636567 -0.24252505 -0.00150322 -0.00208515 -0.00089831 -0.00635749 -0.00448895 0.00468848 0.00260545 -0.00376596 -0.00057884 0.00203802 -0.00856087 -0.00146417 -0.03898270 -0.16470810 0.21965075</matrix>

</property>

<property dictRef="me:frequenciesScaleFactor">

<scalar>1</scalar>

</property>

<property dictRef="me:symmetryNumber">

<scalar>0.5</scalar>

</property>

```
</propertyList>

<me:ExtraDOSCMethod xsi:type="me:HinderedRotorQM1D">
<bondRef>b1</bondRef>
<me:HinderedRotorPotential format="numerical" units="kJ/mol" expansionSize="5" UseSineTerms="yes">
<me:PotentialPoint angle="248.5855594211199" potential="0.0"/>
<me:PotentialPoint angle="258.5855571532413" potential="-0.3074250037316233"/>
<me:PotentialPoint angle="268.5855366907492" potential="2.081837428268045"/>
<me:PotentialPoint angle="278.58563041603844" potential="1.8680377416312695"/>
<me:PotentialPoint angle="288.5855738053534" potential="3.6515816543251276"/>
<me:PotentialPoint angle="298.5856162644453" potential="3.444172433926724"/>
<me:PotentialPoint angle="308.58561515315205" potential="4.380081664770842"/>
<me:PotentialPoint angle="318.5855834188542" potential="4.663218171102926"/>
<me:PotentialPoint angle="328.58560609450524" potential="5.236824204795994"/>
<me:PotentialPoint angle="338.5855585295781" potential="5.4742743918905035"/>
<me:PotentialPoint angle="348.58557104072804" potential="5.801955125527456"/>
<me:PotentialPoint angle="358.58563776103233" potential="5.5344298399286345"/>
<me:PotentialPoint angle="8.585606428559894" potential="5.627831989550032"/>
<me:PotentialPoint angle="18.585599933685764" potential="4.944432812277228"/>
<me:PotentialPoint angle="28.585575406094982" potential="4.447213064879179"/>
<me:PotentialPoint angle="38.58556385112812" potential="3.8234967452008277"/>
<me:PotentialPoint angle="48.58553145446165" potential="3.712582513107918"/>
<me:PotentialPoint angle="58.585634436852324" potential="3.3098938803886995"/>
<me:PotentialPoint angle="68.58558967985303" potential="3.310051410458982"/>
<me:PotentialPoint angle="78.58561773016976" potential="3.6096813055919483"/>
<me:PotentialPoint angle="88.58558223533208" potential="3.9024166389135644"/>
<me:PotentialPoint angle="98.58554224513662" potential="4.279858466936275"/>
<me:PotentialPoint angle="108.58559187554616" potential="4.91619556338992"/>
<me:PotentialPoint angle="118.585598283927" potential="5.08559542556759"/>
<me:PotentialPoint angle="128.58554183367966" potential="5.769020857987925"/>
<me:PotentialPoint angle="138.58555333191993" potential="5.865111519233324"/>
```

```
<me:PotentialPoint angle="148.5855703441631" potential="6.274521430837922"/>
<me:PotentialPoint angle="158.5856310233686" potential="6.29852374875918"/>
<me:PotentialPoint angle="168.58553053554928" potential="6.362000453518704"/>
<me:PotentialPoint angle="178.58552938839125" potential="5.965904450509697"/>
<me:PotentialPoint angle="188.58560256439029" potential="5.713139723753557"/>
<me:PotentialPoint angle="198.5855153228107" potential="5.039615050307475"/>
<me:PotentialPoint angle="208.5855422933186" potential="4.33666902827099"/>
<me:PotentialPoint angle="218.58559376284984" potential="3.6047926254104823"/>
<me:PotentialPoint angle="228.58556474491886" potential="3.322918983991258"/>
<me:PotentialPoint angle="238.58557168711116" potential="2.9312364461366087"/>
   <me:PotentialPoint angle="248.5855594211199" potential="0.0"/>
</me:HinderedRotorPotential>
    <me:CalculateInternalRotorInertia phaseDifference="0"/>
   </me:ExtraDOSCMethod>
<me:ExtraDOSCMethod xsi:type="me:HinderedRotorQM1D">
<bondRef>b2</bondRef>
<me:HinderedRotorPotential format="numerical" units="kJ/mol" expansionSize="5" UseSineTerms="yes">
<me:PotentialPoint angle="180.06190490758095" potential="0.0"/>
<me:PotentialPoint angle="190.044722276271" potential="2.4982994323363528"/>
<me:PotentialPoint angle="200.03827004334136" potential="11.179897310910746"/>
<me:PotentialPoint angle="210.02945230627256" potential="22.671107990317978"/>
<me:PotentialPoint angle="220.02414503525938" potential="37.70558271103073"/>
<me:PotentialPoint angle="230.01938296330889" potential="52.8639652619604"/>
<me:PotentialPoint angle="240.00247485399134" potential="68.09128555678762"/>
<me:PotentialPoint angle="249.99257320982653" potential="84.17656137258746"/>
<me:PotentialPoint angle="259.9836705487136" potential="94.92069663188886"/>
<me:PotentialPoint angle="269.93984102914084" potential="101.33588970825076"/>
<me:PotentialPoint angle="279.9066356208051" potential="140.44818624504842"/>
<me:PotentialPoint angle="289.9186883899843" potential="100.82336592371576"/>
<me:PotentialPoint angle="299.7837979915119" potential="92.451892062556"/>
<me:PotentialPoint angle="309.8606198437122" potential="84.16671574895736"/>
```

```
<me:PotentialPoint angle="319.9562614691991" potential="69.41514123964589"/>
<me:PotentialPoint angle="329.9944425696477" potential="51.95761915715411"/>
<me:PotentialPoint angle="340.0163431077207" potential="32.19037940504495"/>
<me:PotentialPoint angle="350.11842151706276" potential="14.388134471024387"/>
<me:PotentialPoint angle="359.98489061611974" potential="5.89952131791506"/>
<me:PotentialPoint angle="10.017465177183205" potential="5.636112815584056"/>
<me:PotentialPoint angle="19.909713382055045" potential="9.558105179923587"/>
<me:PotentialPoint angle="29.873802371287187" potential="19.387088408577256"/>
<me:PotentialPoint angle="39.76610806530279" potential="29.341969595989212"/>
<me:PotentialPoint angle="49.76770879694637" potential="46.630183708737604"/>
<me:PotentialPoint angle="59.651033141073626" potential="58.11923885287251"/>
<me:PotentialPoint angle="69.73623792863934" potential="83.51781826233491"/>
<me:PotentialPoint angle="79.59574587801504" potential="94.34493243740872"/>
<me:PotentialPoint angle="89.76456298471722" potential="124.74152848136146"/>
<me:PotentialPoint angle="99.73325052948476" potential="136.08461372193415"/>
<me:PotentialPoint angle="109.90128118337367" potential="164.91038645943627"/>
<me:PotentialPoint angle="119.82913861897711" potential="203.10235610604286"/>
<me:PotentialPoint angle="129.97234960883713" potential="145.3130007807631"/>
<me:PotentialPoint angle="139.83909835872578" potential="90.39699751138687"/>
<me:PotentialPoint angle="150.06942348741296" potential="55.944970709620975"/>
<me:PotentialPoint angle="160.0054974525835" potential="28.663075775722973"/>
<me:PotentialPoint angle="170.05741769494665" potential="16.4671573638916"/>
    <me:PotentialPoint angle="180.06190490758095" potential="0.0"/>
</me:HinderedRotorPotential>
    <me:CalculateInternalRotorInertia phaseDifference="0"/>
    </me:ExtraDOSCMethod>
</molecule>


    <molecule id="OOCH2NO2">
  <propertyList>
```

```
   &lt;property title="Energy" dictRef="me:ZPE"&gt;
   &lt;scalar units="kJ/mol"&gt;-126&lt;/scalar&gt;
   &lt;/property&gt;
   &lt;property title="Vibrational Frequencies" dictRef="me:vibFreqs"&gt;
    &lt;array units="cm-1"&gt;36.5724 107.5697 110.1268 162.985 194.5654 210.5406 540.5284 699.1192 781.9472 920.726 1053.4548 1261.2692 1439.7321 1450.9565 1642.9652 1768.4335 3142.5373 3290.3128&lt;/array&gt;
   &lt;/property&gt;
   &lt;property title="Rotational Constants" dictRef="me:rotConsts"&gt;
    &lt;array units="GHz"&gt;7.45177 2.19286 1.79541&lt;/array&gt;
   &lt;/property&gt;
   &lt;property title="Symmetry Number" dictRef="me:symmetryNumber"&gt;
    &lt;scalar&gt;1 &lt;/scalar&gt;
   &lt;/property&gt;
   &lt;property dictRef="me:MW"&gt;
          &lt;scalar units="amu"&gt;92&lt;/scalar&gt;
        &lt;/property&gt;
  &lt;/propertyList&gt;
 &lt;me:energyTransferModel xsi:type="me:ExponentialDown"&gt;
 &lt;me:deltaEDown units="cm-1"&gt;250&lt;/me:deltaEDown&gt;
 &lt;/me:energyTransferModel&gt;
   &lt;me:DOSCMethod&gt;ClassicalRotors&lt;/me:DOSCMethod&gt;
  &lt;/molecule&gt;

&lt;molecule xmlns="http://www.xml-cml.org/schema" spinMultiplicity="2" id="TS2"&gt;
    &lt;atomArray&gt;
    &lt;atom id="a1" elementType="O" hydrogenCount="0" x3="-1.613939" y3="-0.989516" z3="0.079091"/&gt;
    &lt;atom id="a2" elementType="N" hydrogenCount="0" x3="-1.720811 " y3="0.103536" z3="-0.386590"/&gt;
    &lt;atom id="a3" elementType="O" hydrogenCount="0" x3="-0.996380" y3="1.056539" z3="-0.201391"/&gt;
    &lt;atom id="a4" elementType="C" hydrogenCount="2" x3="0.780755" y3="0.336925" z3="0.978924"/&gt;
    &lt;atom id="a5" elementType="O" hydrogenCount="0" x3="1.296591" y3="-0.522811" z3="0.178315"/&gt;
    &lt;atom id="a6" elementType="O" hydrogenCount="0" x3="2.068039" y3="-0.046472" z3="-0.790128"/&gt;
```

<atom id="a7" elementType="H" hydrogenCount="0" x3="1.118099" y3="1.364405" z3="0.914316"/>

<atom id="a8" elementType="H" hydrogenCount="0" x3="0.208562" y3="-0.092621" z3="1.791174"/>

</atomArray>

<bondArray>

<bond atomRefs2="a6 a5" order="1"/>

<bond atomRefs2="a2 a3" order="1" id="b2"/>

<bond atomRefs2="a2 a1" order="2"/>

<bond atomRefs2="a5 a4" order="1" id="b3"/>

<bond atomRefs2="a7 a4" order="1"/>

<bond atomRefs2="a4 a8" order="1"/>

<bond atomRefs2="a4 a3" order="1" id="b1"/>

</bondArray>

<propertyList>

<property dictRef="me:ZPE">

<scalar units="kJ/mol">22</scalar>

</property>

<property dictRef="me:spinMultiplicity">

<scalar units="cm-1">2</scalar>

</property>

<property dictRef="me:symmetryNumber">

<scalar>0.5</scalar>

</property>

<property dictRef="me:hessian">

<matrix matrixType="squareSymmetricLT" rows="24" units="Hartree/Bohr2">

0.04661739 -0.00640040 0.69936346 0.02888721 -0.29273861 0.15289532 -0.04329753 0.05840762 -0.04614379 0.26617225 0.07287077 -0.54403608 0.27351344 0.16094486
0.89500642 -0.05189992 0.26633569 -0.16541329 0.11478925 -0.24219284 0.21518452 -0.02025449 -0.05407480 0.00260790 -0.16547078 -0.21963257 -0.02421781 0.15134499
-0.06150167 -0.15431269 0.02419714 -0.23032263 -0.36196975 -0.03049127 0.28516398 0.53311487 0.00697709 0.02659389 0.00685635 -0.02366126 -0.02155464 -0.03180880
-0.00982879 0.00050099 0.01545154 0.03569090 0.00314843 0.02353188 -0.09380013 -0.02025098 -0.05590109 0.08193907 0.00228174 0.05137118 0.26782433 -0.02664841
0.00034085 -0.02149493 0.06455838 0.01988008 0.03724746 -0.07217499 -0.01943061 -0.03727332 0.15350571 0.66256357 0.00654736 0.00100974 -0.00389620 -0.02211408 -
0.00863693 -0.00105752 0.02007345 0.00223700 0.00055608 -0.30578811 0.02816839 0.53021205 -0.02171262 0.00343584 -0.01192214 0.04525320 0.00228277 0.02367444 -
0.04543679 0.00446262 -0.01942236 -0.05823906 0.03563000 0.08940204 0.20884146 0.02048704 -0.00029763 0.01542552 -0.05044515 -0.00926219 -0.03007145 0.05824932 -
0.00003012 0.03209267 0.01162276 -0.26789159 -0.13135589 0.01034213 0.40382765 0.02264363 -0.00413324 0.01851837 -0.05329721 -0.00316718 -0.03350648 0.04378118
0.00648780 0.02546626 0.03925599 -0.08837622 -0.17130053 -0.21338659 0.03673970 0.34856193 0.00756094 -0.00329934 0.00636627 -0.01603842 0.00157449 -0.01093139
0.01311509 0.00033673 0.00638101 -0.04844662 0.02742698 0.05772413 -0.12005930 -0.07691419 0.12633197 0.15623656 0.00077570 0.00003191 0.00025572 -0.00223665 -

0.00055985 -0.00016077 -0.00114539 0.00175890 -0.00235856 0.01919353 0.03164231 -0.02030210 -0.07446996 -0.10784276 0.08935592 0.05382633 0.07400868 -0.01161680
0.00399354 -0.00890715 0.02624880 -0.00103162 0.01568881 -0.01964135 -0.00233959 -0.00865578 0.07446989 -0.03236651 -0.07188173 0.12228646 0.09025490 -0.18075960
-0.18349240 -0.06354952 0.25134101 -0.00177082 0.00117880 -0.00138267 0.00280469 -0.00016098 0.00146928 -0.00995243 -0.00240926 -0.00464136 -0.07220248 -0.10962017
0.02273978 0.01290544 0.02292201 0.01423505 -0.00203962 0.00101805 -0.00767324 0.06852395 0.00012687 0.00008798 0.00008987 0.00043932 -0.00006337 0.00047099
0.00229795 0.00146328 0.00081654 -0.09542666 -0.31877509 0.03025900 -0.00137081 -0.03042725 -0.00927962 -0.00327037 0.00294567 0.00680991 0.09623124 0.34297490 -
0.00138056 0.00009822 -0.00031867 0.00383959 0.00195029 0.00112474 -0.01061059 -0.00244657 -0.00467553 0.03473206 0.03231629 -0.05915232 -0.00121965 -0.02132878
0.01246356 -0.00485292 0.00074331 -0.00108019 -0.02377745 -0.02715234 0.04599880 -0.00283376 -0.00239615 -0.00194465 0.00437671 0.00237164 0.00301724 -0.00528466
0.00198849 -0.00717551 -0.11276601 -0.07267750 0.13141542 -0.02155232 0.00373609 0.02043597 0.00967137 0.00303840 -0.00058137 0.00173127 0.00097246 0.00326952
0.12665741 0.00029012 -0.00117780 0.00075185 -0.00134574 0.00100474 -0.00113781 0.00131650 -0.00059388 0.00118243 -0.07407453 -0.10832953 0.09862081 0.01968740
0.01192388 -0.02762718 0.00031936 -0.00198486 -0.00177110 -0.00915968 0.00179387 0.01581958 0.06296657 0.09736357 -0.00015800 -0.00115923 0.00026527 0.00033869
0.00111948 -0.00021199 -0.00216399 0.00185449 -0.00319011 0.13832819 0.08177885 -0.22347984 0.01058779 0.00824334 -0.01944350 0.00247332 -0.00398400 0.00425463 -
0.00096939 -0.00201435 0.00563960 -0.14843661 -0.08583858 0.23616595

</matrix>

</property>

<property dictRef="me:frequenciesScaleFactor">

<scalar>1</scalar>

</property>

</propertyList>

<me:ExtraDOSCMethod xsi:type="me:HinderedRotorQM1D">

<bondRef>b1</bondRef>

<me:HinderedRotorPotential format="numerical" units="kJ/mol" expansionSize="10" UseSineTerms="yes">

<me:PotentialPoint angle="61.649766455425265" potential="0.0"/>

<me:PotentialPoint angle="71.6497707355341" potential="-1.5225771251134574"/>

<me:PotentialPoint angle="81.64981698124777" potential="2.461686839815229"/>

<me:PotentialPoint angle="91.64982834465461" potential="1.9290307241026312"/>

<me:PotentialPoint angle="101.6498698023692" potential="4.822058274177834"/>

<me:PotentialPoint angle="111.64979727250011" potential="4.222958638914861"/>

<me:PotentialPoint angle="121.64981409364079" potential="6.258631907403469"/>

<me:PotentialPoint angle="131.64983239527146" potential="5.435133443796076"/>

<me:PotentialPoint angle="141.6497842969692" potential="7.060462122433819"/>

<me:PotentialPoint angle="151.64977578474034" potential="6.231946329004131"/>

<me:PotentialPoint angle="161.64985364344676" potential="7.669643676257692"/>

<me:PotentialPoint angle="171.64981579668685" potential="6.830809669103473"/>

<me:PotentialPoint angle="181.6498198240206" potential="7.891532527166419"/>

<me:PotentialPoint angle="191.64977760844127" potential="6.8944596567889675"/>

<me:PotentialPoint angle="201.64972534807634" potential="7.551015726057813"/>

```
<me:PotentialPoint angle="211.64977285991506" potential="6.5665216244524345"/>
<me:PotentialPoint angle="221.6497953057978" potential="6.907553023775108"/>
<me:PotentialPoint angle="231.6498348029502" potential="5.482174520730041"/>
<me:PotentialPoint angle="241.6497605517806" potential="5.985805738018826"/>
<me:PotentialPoint angle="251.6497928386061" potential="5.0816466744290665"/>
<me:PotentialPoint angle="261.6497889511581" potential="6.2623968737898394"/>
<me:PotentialPoint angle="271.64981946416157" potential="6.511575168231502"/>
<me:PotentialPoint angle="281.64985655251365" potential="9.631514150067233"/>
<me:PotentialPoint angle="291.64977712033397" potential="12.151586850523017"/>
<me:PotentialPoint angle="301.64982001090914" potential="18.556656000437215"/>
<me:PotentialPoint angle="311.6497697104546" potential="22.09848914446775"/>
<me:PotentialPoint angle="321.6498452735534" potential="27.274328243685886"/>
<me:PotentialPoint angle="331.6497865731461" potential="24.808942075120285"/>
<me:PotentialPoint angle="341.64979271678453" potential="23.57524342427496"/>
<me:PotentialPoint angle="351.6497759283049" potential="17.33919869014062"/>
<me:PotentialPoint angle="1.6498750614656263" potential="14.450203907908872"/>
<me:PotentialPoint angle="11.649726334443015" potential="10.30183571355883"/>
<me:PotentialPoint angle="21.64981925854016" potential="9.27679339563474"/>
<me:PotentialPoint angle="31.64985430340482" potential="7.317579927737825"/>
<me:PotentialPoint angle="41.64979827459801" potential="7.082621339592151"/>
<me:PotentialPoint angle="51.6498432309229" potential="6.2676531239412725"/>
    <me:PotentialPoint angle="61.649766455425265" potential="0.0"/>
</me:HinderedRotorPotential>
     <me:CalculateInternalRotorInertia phaseDifference="0"/>
    </me:ExtraDOSCMethod>
<me:ExtraDOSCMethod xsi:type="me:HinderedRotorQM1D">
<bondRef>b2</bondRef>
<me:HinderedRotorPotential format="numerical" units="kJ/mol" expansionSize="10" UseSineTerms="yes">
<me:PotentialPoint angle="6.63406438354096" potential="0.0"/>
<me:PotentialPoint angle="16.63915864335284" potential="-1.0431818949291483"/>
<me:PotentialPoint angle="26.647779690473328" potential="4.6127665704116225"/>
```

```
<me:PotentialPoint angle="36.651223368819124" potential="7.0494455262087286"/>
<me:PotentialPoint angle="46.648055570040924" potential="13.895926802651957"/>
<me:PotentialPoint angle="56.64713306107582" potential="17.24709616310429"/>
<me:PotentialPoint angle="66.6413765649965" potential="23.537307580118068"/>
<me:PotentialPoint angle="76.63800559297037" potential="25.075356774381362"/>
<me:PotentialPoint angle="86.62719768838669" potential="29.463855791138485"/>
<me:PotentialPoint angle="96.62130497491349" potential="29.69955214462243"/>
<me:PotentialPoint angle="106.61387783671614" potential="32.44572762318421"/>
<me:PotentialPoint angle="116.61509190647261" potential="31.516067081829533"/>
<me:PotentialPoint angle="126.61707337868596" potential="32.38918748870492"/>
<me:PotentialPoint angle="136.61913696912336" potential="30.93268106202595"/>
<me:PotentialPoint angle="146.62695422650555" potential="30.918534869910218"/>
<me:PotentialPoint angle="156.6289701290035" potential="29.31992064998485"/>
<me:PotentialPoint angle="166.63101065538245" potential="29.18253613030538"/>
<me:PotentialPoint angle="176.63331784293433" potential="28.02628971892409"/>
<me:PotentialPoint angle="186.6335693844957" potential="28.260106214671396"/>
<me:PotentialPoint angle="196.63315420633253" potential="27.777127177803777"/>
<me:PotentialPoint angle="206.63370184198402" potential="28.666024211794138"/>
<me:PotentialPoint angle="216.63438526689652" potential="28.93558163428679"/>
<me:PotentialPoint angle="226.63757766086908" potential="29.975767834112048"/>
<me:PotentialPoint angle="236.65386617221534" potential="29.844708143384196"/>
<me:PotentialPoint angle="246.65174551279995" potential="30.689050448709168"/>
<me:PotentialPoint angle="256.64424094109125" potential="29.57719598489348"/>
<me:PotentialPoint angle="266.6468642664007" potential="29.49859902798198"/>
<me:PotentialPoint angle="276.6378585031653" potential="27.434662255691364"/>
<me:PotentialPoint angle="286.6387133843822" potential="26.646012048819102"/>
<me:PotentialPoint angle="296.63066928756757" potential="23.79759436310269"/>
<me:PotentialPoint angle="306.6346430595059" potential="21.828125693951733"/>
<me:PotentialPoint angle="316.6311143787251" potential="18.088836598210037"/>
<me:PotentialPoint angle="326.63491466478735" potential="15.41187191568315"/>
<me:PotentialPoint angle="336.6332128842909" potential="11.595647804089822"/>
```

```
<me:PotentialPoint angle="346.6344203982221" potential="9.13276898744516"/>
<me:PotentialPoint angle="356.63348582669806" potential="6.622436890611425"/>
<me:PotentialPoint angle="6.63406438354096" potential="0.0"/>
</me:HinderedRotorPotential>
<me:CalculateInternalRotorInertia phaseDifference="0"/>
</me:ExtraDOSCMethod>
<me:ExtraDOSCMethod xsi:type="me:HinderedRotorQM1D">
<bondRef>b3</bondRef>
<me:HinderedRotorPotential format="numerical" units="kJ/mol" expansionSize="10" UseSineTerms="yes">
<me:PotentialPoint angle="350.38144916049134" potential="0.0"/>
<me:PotentialPoint angle="0.48638838561308434" potential="0.2631407013395801"/>
<me:PotentialPoint angle="10.59312102516287" potential="9.902284553041682"/>
<me:PotentialPoint angle="20.662910184438896" potential="17.77590592193883"/>
<me:PotentialPoint angle="30.62156428212205" potential="33.67800062755123"/>
<me:PotentialPoint angle="40.71952448118727" potential="46.61297093308531"/>
<me:PotentialPoint angle="50.651433024580605" potential="64.51283456920646"/>
<me:PotentialPoint angle="60.74469442875526" potential="78.04294263059273"/>
<me:PotentialPoint angle="70.7100821489505" potential="90.79811872076243"/>
<me:PotentialPoint angle="80.5058010920952" potential="98.1927124292124"/>
<me:PotentialPoint angle="91.17771763534085" potential="93.94599564396776"/>
<me:PotentialPoint angle="100.62865340092534" potential="94.88420218683314"/>
<me:PotentialPoint angle="110.54800223093844" potential="84.44689069199376"/>
<me:PotentialPoint angle="120.39377880464025" potential="67.23360571346711"/>
<me:PotentialPoint angle="130.38993579860747" potential="51.576548888580874"/>
<me:PotentialPoint angle="140.42066147780946" potential="38.292153461952694"/>
<me:PotentialPoint angle="150.43375821392527" potential="27.163112079142593"/>
<me:PotentialPoint angle="160.45793613168723" potential="19.955926534719765"/>
<me:PotentialPoint angle="170.45941752001337" potential="13.74368195515126"/>
<me:PotentialPoint angle="180.44028300619527" potential="13.435899883275852"/>
<me:PotentialPoint angle="190.457196396965" potential="13.566074780886993"/>
<me:PotentialPoint angle="200.42313154596295" potential="21.286303958506323"/>
```

```
&lt;me:PotentialPoint angle="210.47742847255276" potential="30.91149065445643"/&gt;
&lt;me:PotentialPoint angle="220.4087365534245" potential="48.50401067745406"/&gt;
&lt;me:PotentialPoint angle="230.46870073485064" potential="68.65872954274528"/&gt;
&lt;me:PotentialPoint angle="240.42065703642862" potential="90.4376218674006"/&gt;
&lt;me:PotentialPoint angle="250.44291747230412" potential="111.25706735660788"/&gt;
&lt;me:PotentialPoint angle="260.44877245861153" potential="124.62050607532728"/&gt;
&lt;me:PotentialPoint angle="270.9246548232789" potential="127.43577157706022"/&gt;
&lt;me:PotentialPoint angle="280.6970084126812" potential="113.79398788860999"/&gt;
&lt;me:PotentialPoint angle="290.68790008252427" potential="90.45137423428241"/&gt;
&lt;me:PotentialPoint angle="300.52552283566996" potential="70.39567083667498"/&gt;
&lt;me:PotentialPoint angle="310.50083944757324" potential="50.6281685346039"/&gt;
&lt;me:PotentialPoint angle="320.4399471796961" potential="36.082540842471644"/&gt;
&lt;me:PotentialPoint angle="330.5009704301126" potential="25.715217291377485"/&gt;
&lt;me:PotentialPoint angle="340.7609824740177" potential="17.815894906641915"/&gt;
&lt;me:PotentialPoint angle="350.38144916049134" potential="0.0"/&gt;
&lt;/me:HinderedRotorPotential&gt;
&lt;me:CalculateInternalRotorInertia phaseDifference="0"/&gt;
&lt;/me:ExtraDOSCMethod&gt;
&lt;/molecule&gt;

&lt;molecule id="OOCH2ONO"&gt;
&lt;propertyList&gt;
&lt;property title="Energy" dictRef="me:ZPE"&gt;
&lt;scalar units="kJ/mol"&gt;-156&lt;/scalar&gt;
&lt;/property&gt;
&lt;property title="Vibrational Frequencies" dictRef="me:vibFreqs"&gt;
&lt;array units="cm-1"&gt;36.5724 107.5697 110.1268 162.985 194.5654 210.5406 540.5284 699.1192 781.9472 920.726 1053.4548 1261.2692 1439.7321 1450.9565 1642.9652 1768.4335 3142.5373 3290.3128&lt;/array&gt;
&lt;/property&gt;
&lt;property title="Rotational Constants" dictRef="me:rotConsts"&gt;
```

```
    <array units="GHz">7.45177 2.19286 1.79541</array>
   </property>
   <property title="Symmetry Number" dictRef="me:symmetryNumber">
    <scalar>1 </scalar>
   </property>
   <property dictRef="me:MW">
          <scalar units="amu">92</scalar>
        </property>
  </propertyList>
 <me:energyTransferModel xsi:type="me:ExponentialDown">
 <me:deltaEDown units="cm-1">250</me:deltaEDown>
 </me:energyTransferModel>
   <me:DOSCMethod>ClassicalRotors</me:DOSCMethod>
  </molecule>

<molecule id="TS6">
 <propertyList>
  <property dictRef="me:ZPE">
   <scalar units="kJ/mol">-86</scalar>
  </property>
  <property dictRef="me:rotConsts">
   <array units="GHz"> 5.5119500 3.3180200 2.2246400 </array>
  </property>
  <property dictRef="me:vibFreqs">
   <array units="cm-1"> 128.2076 164.4839 177.3422 301.3451 345.3390 419.2101 499.9614 541.4747 836.4649 1127.2150 1238.2106 1342.1804 1465.5587 1623.9678
2022.4283 3019.7531 3111.5250 </array>
  </property>
  <property title="ImaginaryFrequency" dictRef="me:imFreqs">
   <array units="cm-1"> 532.2821 </array>
  </property>
  <property dictRef="me:frequenciesScaleFactor">
```

```
  <scalar>1</scalar>
 </property>
 <property dictRef="me:symmetryNumber">
  <scalar>1</scalar>
 </property>
 <property dictRef="me:MW">
  <scalar units="amu">92</scalar>
 </property>
 <property dictRef="me:spinMultiplicity">
  <scalar>3</scalar>
 </property>
</propertyList>
<me:DOSCMethod name="ClassicalRotors"/>
</molecule>

   <molecule id="HCHO">
  <propertyList>
  <property title="Energy" dictRef="me:ZPE">
  <scalar units="kJ/mol">-75</scalar>
  </property>
  <property title="Vibrational Frequencies" dictRef="me:vibFreqs">
   <array units="cm-1">1213.6323 1273.5787 1540.0681 1870.2951 2945.3436 3015.4336</array>
  </property>
  <property title="Rotational Constants" dictRef="me:rotConsts">
   <array units="GHz">284.78174 39.46619 34.66252</array>
  </property>
  <property title="Symmetry Number" dictRef="me:symmetryNumber">
   <scalar>1 </scalar>
  </property>
  <property dictRef="me:MW">
        <scalar units="amu">30</scalar>
```

```
        &lt;/property&gt;
  &lt;/propertyList&gt;
 &lt;me:energyTransferModel xsi:type="me:ExponentialDown"&gt;
 &lt;me:deltaEDown units="cm-1"&gt;250&lt;/me:deltaEDown&gt;
 &lt;/me:energyTransferModel&gt;
   &lt;me:DOSCMethod&gt;ClassicalRotors&lt;/me:DOSCMethod&gt;
  &lt;/molecule&gt;

     &lt;molecule id="NO"&gt;
   &lt;propertyList&gt;
   &lt;property title="Energy" dictRef="me:ZPE"&gt;
   &lt;scalar units="kJ/mol"&gt;-50&lt;/scalar&gt;
   &lt;/property&gt;
   &lt;property title="Vibrational Frequencies" dictRef="me:vibFreqs"&gt;
    &lt;array units="cm-1"&gt; 2066.0389&lt;/array&gt;
   &lt;/property&gt;
   &lt;property title="Rotational Constants" dictRef="me:rotConsts"&gt;
    &lt;array units="GHz"&gt;0 52.5361972 52.5361972&lt;/array&gt;
   &lt;/property&gt;
   &lt;property title="Symmetry Number" dictRef="me:symmetryNumber"&gt;
    &lt;scalar&gt;1 &lt;/scalar&gt;
   &lt;/property&gt;
   &lt;property dictRef="me:MW"&gt;
          &lt;scalar units="amu"&gt;30&lt;/scalar&gt;
        &lt;/property&gt;
  &lt;/propertyList&gt;
 &lt;me:energyTransferModel xsi:type="me:ExponentialDown"&gt;
 &lt;me:deltaEDown units="cm-1"&gt;250&lt;/me:deltaEDown&gt;
 &lt;/me:energyTransferModel&gt;
   &lt;me:DOSCMethod&gt;ClassicalRotors&lt;/me:DOSCMethod&gt;
  &lt;/molecule&gt;
```

```
  <molecule id="O2">
   <propertyList>
   <property title="Energy" dictRef="me:ZPE">
   <scalar units="kJ/mol">-50</scalar>
   </property>
   <property title="Vibrational Frequencies" dictRef="me:vibFreqs">
    <array units="cm-1">1754.4088</array>
   </property>
   <property title="Rotational Constants" dictRef="me:rotConsts">
    <array units="GHz">0 44.6349128 44.6349128</array>
   </property>
   <property title="Symmetry Number" dictRef="me:symmetryNumber">
    <scalar>1 </scalar>
   </property>
   <property dictRef="me:MW">
          <scalar units="amu">32</scalar>
        </property>
  </propertyList>
 <me:energyTransferModel xsi:type="me:ExponentialDown">
 <me:deltaEDown units="cm-1">250</me:deltaEDown>
 </me:energyTransferModel>
   <me:DOSCMethod>ClassicalRotors</me:DOSCMethod>
  </molecule>

<molecule id="N2">
 <atom elementType="N"/>
 <propertyList>
  <property dictRef="me:epsilon">
   <scalar>48.0</scalar>
  </property>
```

```
  &lt;property dictRef="me:sigma"&gt;
   &lt;scalar&gt;3.9&lt;/scalar&gt;
  &lt;/property&gt;
  &lt;property dictRef="me:MW"&gt;
   &lt;scalar units="amu"&gt;28.0&lt;/scalar&gt;
  &lt;/property&gt;
 &lt;/propertyList&gt;
&lt;/molecule&gt;

&lt;/moleculeList&gt;

&lt;reactionList&gt;
       &lt;reaction id="R1"&gt;
     &lt;reactantList&gt;
      &lt;reactant&gt;
       &lt;molecule ref="CH2OO" role="deficientReactant"/&gt;
      &lt;/reactant&gt;
      &lt;reactant&gt;
       &lt;molecule ref="NO2" role="excessReactant"/&gt;
      &lt;/reactant&gt;
     &lt;/reactantList&gt;
     &lt;productList&gt;
      &lt;product&gt;
       &lt;molecule ref="prc" role="modelled"/&gt;
      &lt;/product&gt;
     &lt;/productList&gt;
     &lt;me:MCRCMethod xsi:type="me:MesmerILT"&gt;
                &lt;me:preExponential units="cm3molecule-1s-1" lower="1e-13" upper="1e-10" stepsize="1e-13"&gt;1.00e-12&lt;/me:preExponential&gt;
         &lt;me:activationEnergy  units="cm-1"&gt;0.0&lt;/me:activationEnergy&gt;
         &lt;me:nInfinity&gt;-1.49&lt;/me:nInfinity&gt;
       &lt;/me:MCRCMethod&gt;
```

```
        &lt;me:excessReactantConc&gt;1.0E16&lt;/me:excessReactantConc&gt;
    &lt;/reaction&gt;

&lt;reaction id="R2"&gt;
 &lt;reactantList&gt;
     &lt;reactant&gt;
      &lt;molecule ref="prc" role="modelled"/&gt;
     &lt;/reactant&gt;
 &lt;/reactantList&gt;
   &lt;me:transitionState&gt;
  &lt;molecule ref="TS1" me:type="transitionState" /&gt;
 &lt;/me:transitionState&gt;
 &lt;productList&gt;
  &lt;product&gt;
   &lt;molecule ref="OOCH2NO2" role="modelled"/&gt;
  &lt;/product&gt;
 &lt;/productList&gt;
 &lt;me:MCRCMethod name="SimpleRRKM"/&gt;
 &lt;/reaction&gt;

  &lt;reaction id="R3"&gt;
 &lt;reactantList&gt;
     &lt;reactant&gt;
      &lt;molecule ref="prc" role="modelled"/&gt;
     &lt;/reactant&gt;
 &lt;/reactantList&gt;
   &lt;me:transitionState&gt;
  &lt;molecule ref="TS2" me:type="transitionState" /&gt;
 &lt;/me:transitionState&gt;
 &lt;productList&gt;
  &lt;product&gt;
```

```
    <molecule ref="OOCH2ONO" role="modelled"/>
   </product>
  </productList>
  <me:MCRCMethod name="SimpleRRKM"/>
  </reaction>

   <reaction id="R4">
  <reactantList>
   <reactant>
    <molecule ref="OOCH2ONO" role="modelled"/>
   </reactant>
  </reactantList>
  <productList>
   <product>
    <molecule ref="HCHO" role="sink"/>
   </product>
     <product>
    <molecule ref="NO" role="sink"/>
   </product>
     <product>
    <molecule ref="O2" role="modelled"/>
   </product>
  </productList>
     <me:transitionState>
   <molecule ref="TS6" role="transitionState"/>
  </me:transitionState>
        <me:MCRCMethod name="SimpleRRKM"/>
 </reaction>

</reactionList>
```

```
<me:conditions>

 <me:bathGas>N2</me:bathGas>

 <me:PTs>

   <me:PTpair units="Torr" P= "50" T="223" precision="dd"> <me:bathGas>N2</me:bathGas><me:experimentalEigenvalue EigenvalueID="3" error="1.97E3" >
  1.97E4  </me:experimentalEigenvalue> </me:PTpair>

   <me:PTpair units="Torr" P= "25" T="242" precision="dd"> <me:bathGas>N2</me:bathGas><me:experimentalEigenvalue EigenvalueID="3" error="1.83E3" >
  1.83E4  </me:experimentalEigenvalue> </me:PTpair>

   <me:PTpair units="Torr" P= "50" T="242" precision="dd"> <me:bathGas>N2</me:bathGas><me:experimentalEigenvalue EigenvalueID="3" error="1.85E3" >
  1.85E4  </me:experimentalEigenvalue> </me:PTpair>

   <me:PTpair units="Torr" P= "200" T="242" precision="dd"> <me:bathGas>N2</me:bathGas><me:experimentalEigenvalue EigenvalueID="3" error="1.86E3"       >
  1.86E4  </me:experimentalEigenvalue> </me:PTpair>

   <me:PTpair units="Torr" P= "25" T="254" precision="dd"> <me:bathGas>N2</me:bathGas><me:experimentalEigenvalue EigenvalueID="3" error="1.65E3" >
  1.65E4  </me:experimentalEigenvalue> </me:PTpair>

   <me:PTpair units="Torr" P= "50" T="254" precision="dd"> <me:bathGas>N2</me:bathGas><me:experimentalEigenvalue EigenvalueID="3" error="1.70E3" >
  1.70E4  </me:experimentalEigenvalue> </me:PTpair>

   <me:PTpair units="Torr" P= "200" T="254" precision="dd"> <me:bathGas>N2</me:bathGas><me:experimentalEigenvalue EigenvalueID="3" error="1.41E3"       >
  1.41E4  </me:experimentalEigenvalue> </me:PTpair>

   <me:PTpair units="Torr" P= "50" T="273" precision="dd"> <me:bathGas>N2</me:bathGas><me:experimentalEigenvalue EigenvalueID="3" error="1.31E3" >
  1.31E4  </me:experimentalEigenvalue> </me:PTpair>

   <me:PTpair units="Torr" P= "50" T="277" precision="dd"> <me:bathGas>N2</me:bathGas><me:experimentalEigenvalue EigenvalueID="3" error="1.37E3" >
  1.37E4  </me:experimentalEigenvalue> </me:PTpair>

   <me:PTpair units="Torr" P= "25" T="298" precision="dd"> <me:bathGas>N2</me:bathGas><me:experimentalEigenvalue EigenvalueID="3" error="1.35E3" >
  1.35E4  </me:experimentalEigenvalue> </me:PTpair>

   <me:PTpair units="Torr" P= "50" T="298" precision="dd"> <me:bathGas>N2</me:bathGas><me:experimentalEigenvalue EigenvalueID="3" error="1.11E3" >
  1.11E4  </me:experimentalEigenvalue> </me:PTpair>

   <me:PTpair units="Torr" P= "100" T="298" precision="dd"> <me:bathGas>N2</me:bathGas><me:experimentalEigenvalue EigenvalueID="3" error="1.26E3"       >
  1.26E4  </me:experimentalEigenvalue> </me:PTpair>

   <me:PTpair units="Torr" P= "200" T="298" precision="dd"> <me:bathGas>N2</me:bathGas><me:experimentalEigenvalue EigenvalueID="3" error="1.41E3"       >
  1.41E4  </me:experimentalEigenvalue> </me:PTpair>

   <me:PTpair units="Torr" P= "300" T="298" precision="dd"> <me:bathGas>N2</me:bathGas><me:experimentalEigenvalue EigenvalueID="3" error="1.18E3"       >
  1.18E4  </me:experimentalEigenvalue> </me:PTpair>

   <me:PTpair units="Torr" P= "50" T="318" precision="dd"> <me:bathGas>N2</me:bathGas><me:experimentalEigenvalue EigenvalueID="3" error="9.57E2" >
  9.57E3  </me:experimentalEigenvalue> </me:PTpair>

   <me:PTpair units="Torr" P= "50" T="323" precision="dd"> <me:bathGas>N2</me:bathGas><me:experimentalEigenvalue EigenvalueID="3" error="8.39E2" >
  8.39E3  </me:experimentalEigenvalue> </me:PTpair>

   <me:PTpair units="Torr" P= "50" T="335" precision="dd"> <me:bathGas>N2</me:bathGas><me:experimentalEigenvalue EigenvalueID="3" error="7.79E2" >
  7.79E3  </me:experimentalEigenvalue> </me:PTpair>

   <me:PTpair units="Torr" P= "50" T="353" precision="dd"> <me:bathGas>N2</me:bathGas><me:experimentalEigenvalue EigenvalueID="3" error="5.77E2" >
  5.77E3  </me:experimentalEigenvalue> </me:PTpair>
```

```
        &lt;me:PTpair units="Torr" P= "50" T="363" precision="dd"&gt; &lt;me:bathGas&gt;N2&lt;/me:bathGas&gt;&lt;me:experimentalEigenvalue EigenvalueID="3" error="5.98E2" &gt;
       5.98E3  &lt;/me:experimentalEigenvalue&gt; &lt;/me:PTpair&gt;

 &lt;/me:PTs&gt;
&lt;/me:conditions&gt;

&lt;me:modelParameters&gt;
 &lt;me:grainSize units="cm-1"&gt;50&lt;/me:grainSize&gt;
 &lt;me:energyAboveTheTopHill&gt;25.0&lt;/me:energyAboveTheTopHill&gt;
&lt;/me:modelParameters&gt;

&lt;me:control&gt;
 &lt;me:calcMethod xsi:type="me:marquardt"&gt;
  &lt;me:MarquardtIterations&gt;10&lt;/me:MarquardtIterations&gt;
  &lt;me:MarquardtTolerance&gt;1e-8&lt;/me:MarquardtTolerance&gt;
  &lt;me:MarquardtDerivDelta&gt;0.025&lt;/me:MarquardtDerivDelta&gt;
 &lt;/me:calcMethod&gt;
 &lt;me:printSpeciesProfile /&gt;
 &lt;me:printGrainkfE /&gt;
 &lt;me:eigenvalues&gt;5&lt;/me:eigenvalues&gt;
&lt;/me:control&gt;

&lt;/me:mesmer&gt;
```
